# Molecular Basis for DNA Double-Strand Break Annealing and Primer Extension by an NHEJ DNA Polymerase

**DOI:** 10.1016/j.celrep.2013.10.016

**Published:** 2013-11-14

**Authors:** Nigel C. Brissett, Maria J. Martin, Edward J. Bartlett, Julie Bianchi, Luis Blanco, Aidan J. Doherty

**Affiliations:** 1 Genome Damage and Stability Centre, University of Sussex, Brighton BN1 9RQ, UK; 2 Centro de Biología Molecular Severo Ochoa, CSIC-UAM, 28049 Madrid, Spain

## Abstract

Nonhomologous end-joining (NHEJ) is one of the major DNA double-strand break (DSB) repair pathways. The mechanisms by which breaks are competently brought together and extended during NHEJ is poorly understood. As polymerases extend DNA in a 5′-3′ direction by nucleotide addition to a primer, it is unclear how NHEJ polymerases fill in break termini containing 3′ overhangs that lack a primer strand. Here, we describe, at the molecular level, how prokaryotic NHEJ polymerases configure a primer-template substrate by annealing the 3′ overhanging strands from opposing breaks, forming a gapped intermediate that can be extended in *trans*. We identify structural elements that facilitate docking of the 3′ ends in the active sites of adjacent polymerases and reveal how the termini act as primers for extension of the annealed break, thus explaining how such DSBs are extended in *trans*. This study clarifies how polymerases couple break-synapsis to catalysis, providing a molecular mechanism to explain how primer extension is achieved on DNA breaks.

## Highlights

• Structure of a NHEJ polymerase bound to an annealed DNA double-strand break
• Break synapsis is stabilized by microhomology and polymerase surface loops
• 3′ hydroxyl of the primer strand is positioned into active-site pocket in *trans*
• Templating base selection relies on loop 1 and conserved phenylalanine residues

## Graphical Abstract

**Figure:**
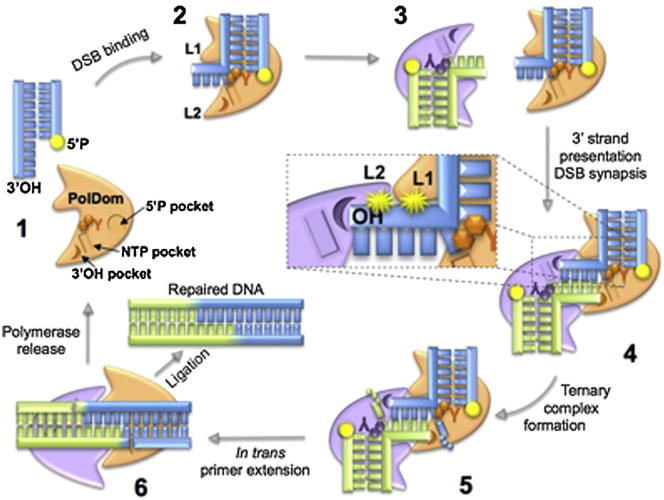

## Teaser

In this article, Brissett and colleagues have elucidated the molecular basis for the extension of the termini of a synapsed DNA double-strand break by a mycobacterial NHEJ repair polymerase. This structure captures the moments after the break has been annealed back together, revealing how the broken ends are positioned into the active site of adjacent polymerases in readiness for filling in of the remaining gaps to, ultimately, facilitate rejoining of the break by DNA ligase.

## Introduction

Double-strand breaks (DSBs) are among the most catastrophic DNA lesions encountered by cells and efficient repair is necessary to prevent genomic instability. Two major cellular pathways have evolved to repair DSBs in organisms from prokaryotes to eukaryotes (Chapman et al., 2012). Homologous recombination (HR) offers error-free repair of breaks, utilizing a sister chromatid as a template to replicate lost genetic material. In noncycling cells, nonhomologous end-joining (NHEJ) allows the direct reconnection of severed DNA termini without the requirement for a template. NHEJ of noncomplementary DNA breaks requires end processing and, as a consequence of operating without a template, is considered to be more error prone than HR.

NHEJ in higher eukaryotes is principally conducted by the Ligase IV, XRCC4 and XLF (LXX) complex, DNA-PKcs, and Ku 70/80 (Daley et al., 2005a; Mahaney et al., 2009). Ku binds to and preserves the broken ends and, with DNA-PKcs, enforces proximity of the break termini. Ku recruits LXX complex, enabling the ligation of the DNA. Damaged or incompatible ends require remodeling prior to ligation, tasks accomplished by a variety of processing enzymes, notably DNA polymerases μ and λ, PNKP (polynucleotide kinase and phosphatase) and Artemis. Prokaryotes, and some archaeal species, possess a more minimal yet functionally analogous NHEJ complex, consisting of Ligase D (LigD) and a Ku homodimer (Weller et al., 2002; Della et al., 2004; Gong et al., 2005; Pitcher et al., 2005, 2007a; Bartlett et al., 2013). Mycobacterial LigD comprises three distinct enzymatic domains: DNA ligase, phosphoesterase (PE), and a polymerase (PolDom). However, many species encode these activities on distinct genes (Bartlett et al., 2013). PE and PolDom of LigD remodel incompatible DNA termini for ligation. In many organisms, Ku and LigD form an NHEJ complex that is capable of repairing a wide variety of DSBs that arise in stationary or sporulation stages of the cell cycle (Weller et al., 2002; Moeller et al., 2007; Pitcher et al., 2007c). NHEJ has also been shown to be required for genome circularization of some mycobacterial phage (Pitcher et al., 2006).

Archaeo-prokaryotic (AP) NHEJ polymerases (PolDom or LigD Pol) are members of the archaeo-eukaryotic primase (AEP) superfamily (Aravind and Koonin, 2001; Weller and Doherty, 2001; Iyer et al., 2005; Bartlett et al., 2013). AEPs encompass a broad family of primordial polymerases that have recently been appreciated to have more diverse roles in DNA replication and repair (Della et al., 2004; Zhu and Shuman, 2005; Zhu et al., 2006; Pitcher et al., 2007b; Bartlett et al., 2013). Structural studies have revealed that AEPs possess an open active site that displays reduced template dependency and greater catalytic flexibility to allow varied substrate interactions (Brissett et al., 2007; Pitcher et al., 2007b). The ability to bypass lesions, distort templates, and displace strands facilitates the roles of these NHEJ polymerases in repairing DSBs. Conventional DNA polymerases extend off double-stranded DNA (dsDNA) substrates, containing both primer and template strands, in a 5′ to 3′ direction. In contrast, polymerases involved in DSB repair must be capable of binding to and extending off noncanonical DNA polymerase substrates, including 3′ overhanging termini, lacking continuous primer and template strands (Brissett et al., 2007, 2011).

Although recent studies have provided insights into AP-NHEJ polymerase-mediated orchestration of break synapsis (Brissett et al., 2007), the order of substrate binding events and mechanisms by which these NHEJ polymerases catalyze end extension is still poorly understood. Here, we describe a polymerase-DNA complex that represents a crucial step in the NHEJ repair process, the productive bridging of two DNA ends to form a microhomology-mediated annealed break. The 3′ overhangs are positioned in an in *trans* configuration in the active site of an adjacent polymerase in readiness for extension. We demonstrate that two conserved surface loops (loop 1 and 2) play critical roles in facilitating this process, acting as molecular chaperones that promote break annealing, and guide the incoming primer strands into the active sites of the neighboring polymerases. Finally, we identify a number of conserved active-site residues that assist in docking the 3′ hydroxyl in a catalytically competent location, awaiting the arrival of incoming nucleotide and metal ions to repair the break.

## Results

### A Functional NHEJ Polymerase-Mediated Synapsis

**Figure 1:**
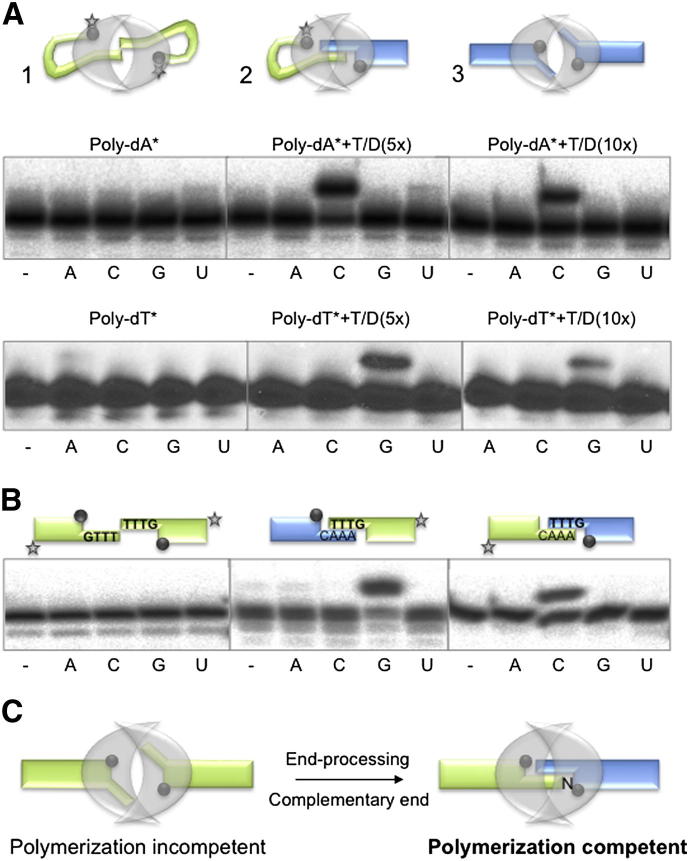
A Functional NHEJ Polymerase-Mediated Synapsis (A) NHEJ reactions were performed with PolDom (600 nM) using a homopolymeric single-stranded DNA substrate (poly-dA or poly-dT) and a 3′-protruding substrate formed with the oligonucleotides TTTG or AAAC and NHEJ-D. In this and the other figures, the black spheres indicate the presence of a 5′-P group in the substrate and the star denotes the position of the radioactive label. When indicated, each of the four NTPs (100 μM) were added in the presence of 1 mM MnCl_2_. (B) NHEJ reactions were performed with PolDom (600 nM) using DNA substrates formed with the oligonucleotides TTTG with NHEJ-D and AAAC with NHEJ-D2. When indicated, each of the four NTPs (100 μM) was added in the presence of 1 mM MnCl_2_. (C) A stable dimeric complex formed at noncomplementary DNA ends (polymerization incompetent) would allow further nucleolytic resection to produce a polymerization competent DNA substrate. See also Figure S1.

Although AP-NHEJ polymerases are members of the AEP superfamily, they are preferentially template-directed DNA polymerases (Della et al., 2004; Pitcher et al., 2007b; Bartlett et al., 2013). Using a single-stranded homopolymer (Poly-dA) as the DNA substrate, we observed that *Mt*-PolDom catalyzed preferential extension with a single nucleotide (UTP), but this reaction was highly inefficient (Figure 1A, left), Consistently, ATP was preferentially inserted when poly-dT was the template, again complementary to the homopolymer (Figure 1A, bottom). These data indicate that nucleotide incorporation does not occur via terminal transferase activity but is the result of in *trans* extension, directed by a second DNA introduced by a synaptic arrangement of the DNA termini (Figure 1A, scheme 1). This arrangement is promoted by a specific interaction of each polymerase with a 5′-P moiety as previously proposed (Brissett et al., 2007).

The inefficiency of this reaction is likely to be the result of a noncomplementary synapsis, in which the primer terminus would be unpaired in the vicinity of the nucleotide binding site. In contrast, by adding a 5-fold excess of an unlabeled template/downstream (T/D) molecule with a 3′ protrusion of 4 nt (GTTT-3′) and a recessed 5′-phosphate, the nucleotide preferentially inserted into the labeled Poly-dA was CTP, complementary to dG next to the 5′-P of the T/D molecule (Figure 1A, center). In this case, both the synapsis and precise approach of the 3′ terminus (Poly-dA) into the active site is facilitated by the complementarity of the last three 3′-terminal bases of each molecule (AAA-3′/TTT-3′). Notably, addition of even higher amounts of T/D (GTTT-3′) DNA (∼10-fold excess over Poly-dA) significantly inhibited extension of Poly-dA with CTP (Figure 1A, right). These experiments were repeated using labeled Poly-dT and a cold 3′-protruding substrate (CAAA-3′, Figure 1A, bottom). These data suggest that the unlabeled DNA is competing for PolDom binding, and this could lead to a stable synaptic arrangement of two T/D molecules (nonlabeled, see Figure 1A, scheme 3) similar to that observed in the structure of an Mt-PolDom synapsis with two DNA ends forming an imperfect DNA synapsis (Brissett et al., 2007). This kind of imperfect, but stable, synapsis is catalytically incompetent as extension does not occur on labeled T/D (GTTT-3′) DNA upon addition of each of the four NTPs (Figure 1B, left). Conversely, when two compatible ends (GTTT-3′/CAAA-3′) were simultaneously present, each 3′-protruding end could be preferentially extended with the nucleotide complementary to the templating base neighboring the 5′-P, provided in *trans* by the opposite end (Figure 1B, middle and right), as expected for bona fide NHEJ reactions. Similar results were obtained with T/D molecules having shorter protrusions (Figure S1). From these data, it can be inferred that a stable synaptic complex is formed, as depicted in Figure 1C, even when the two 3′-protruding ends are not complementary, perhaps to allow further nucleolytic resection to occur in order to produce a polymerization-competent DNA substrate.

### Crystal Structure of an In *trans* Configured Polymerase-DNA Synaptic Complex

**Figure 2:**
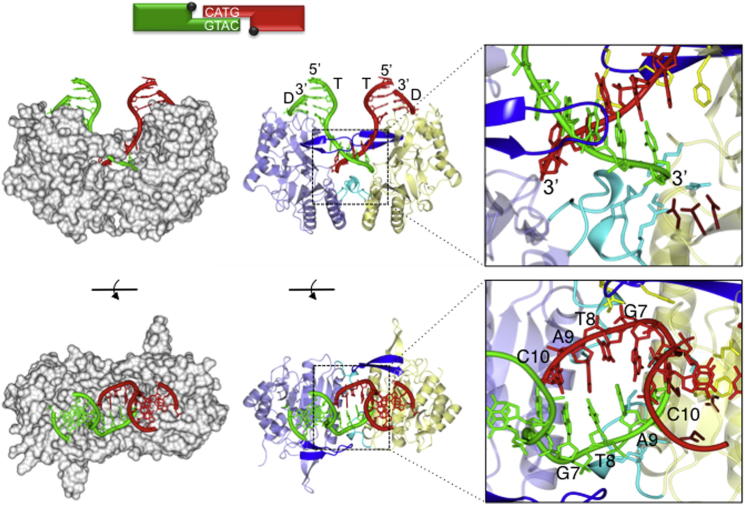
Architecture of an Annealed dsDNA Break Bound to an NHEJ Polymerase Schematic representation of the annealed DNA double-strand break present in the crystal structure with the annealed microhomology sequence highlighted. Below this scheme are representations of the crystal structure of the annealed DNA double-strand break bound to an NHEJ polymerase, PolDom. The figure depicts a synaptic complex formed between two binary (DNA [T/D]-PolDom) complexes that have come together, in a “face-to-face” orientation, by annealing of the 3′ self-complementary DNA overhangs of the break. To the left of the figure, the polymerase is depicted as a gray solvent accessible surface, and the DNA is depicted in red or green (side-on and top-down views). The polymerases facilitate DNA break synapsis between discontinuous DNA ends by cradling the termini, within a continuous molecular surface, promoting microhomology-mediated end synapsis. The middle of the figure has a protein ribbon representation of the structure of the annealed DNA break bound to PolDom (side-on and top-down views). The polymerase monomers are colored light blue and yellow, respectively. Significant structural elements loop 1 and loop 2 are colored blue and cyan, respectively. The polymerase induces a major splaying (∼119**°)** of the template strand (T). The resulting 3′ overhangs are annealed together, forming four Watson-Crick base pairs (G7-C10), via a region of microhomology. This end synapsis is promoted by interactions with loops 1 and 2 (inset). The template strand from one binary complex terminates in the active site of the opposing binary complex, effectively becoming an incoming primer strand (inset). The inset also features catalytic site residues (tan) as well as residues involved in template strand splaying and orientation (yellow) and primer strand orientation and tethering (cyan) (see Figures 3A, 6A, 6B, 7A, 7B, and S4–S7 for more detail). See also Figure S2.

To understand the molecular basis for the proposed in *trans* templated polymerization extensions, PolDom was crystallized in complex with dsDNA containing a self-complementary 3′ overhang. The DNA substrate consisted of a template (T) strand (ten bases) annealed to a recessed downstream (D) strand (5 bases), resulting in a 5 bp 3′ overhang on the T strand (Figure 2). Crystallization and structure determination are described in the Experimental Procedures and Supplemental Information. The complex consists of two PolDom monomers (residues 10–293), each bound to a DNA “end” forming two PolDom-DNA binary complexes. The binary complexes are brought together by a continuous molecular cradle, formed by the polymerases, that promotes synapsis between the discontinuous DNA termini (Figure 2). The 3′-overhanging template strands are further stabilized by a region of microhomology, formed by four Watson-Crick base pairs (G7-C10; Figure 2). PolDom monomers face one another, with the bound duplexes (T/D) appearing in a near parallel orientation on the top of the complex. PolDom interacts with the recessed 5′ phosphate of the downstream strand, noted previously (Brissett et al., 2007, 2011) and splays the templating strand at the ds/ss junction by ∼119°. The templating strand from one binary complex appears to terminate in the active site of the opposing PolDom monomer. This configuration effectively makes the outgoing “templating” strand from one binary complex an incoming “primer” strand for the other monomer and exemplifies the term in *trans* for this complex. The DNA duplex regions are on the same side of the complex (Figures 2 and S2A). The orientation of the PolDom monomers is in dramatic contrast to that observed in the imperfect PolDom-DNA synaptic complex (PDB: 2R9L), in which the PolDom monomers adopt an orientation where the duplex regions are on opposite sides of the complex (Figure S2B). The protein monomers are rotated by ∼180° with respect to one another. This orientation facilitates a near catalytically competent end-synapsis configuration that positions the 3′ primer strand in the active site of the adjacent polymerase to permit extension to occur in *trans*, discussed below.

### Formation of Functional NHEJ Complexes on Short Overhangs: Role of 5′ Phosphate Binding and Synaptic versus Monomeric Configurations

**Figure 3:**
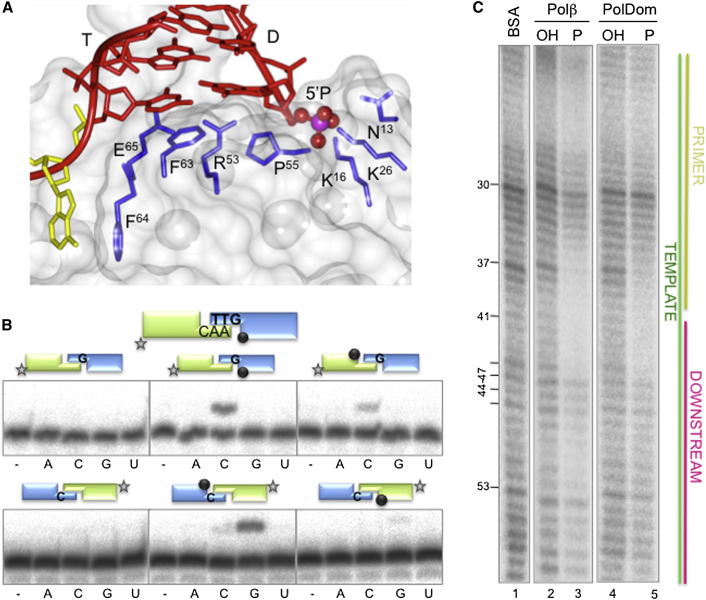
Formation of Functional NHEJ Complexes on Short Overhangs: Role of 5′ Phosphate Binding and Dimeric versus Monomeric Configurations (A) Schematic representation of the phosphate binding region and DNA ds/ss (T/D) junction of annealed break DNA bound to PolDom. The protein is depicted as a translucent solvent accessible surface and DNA (red) is depicted with protein side-chain neighbors that are within 4 Å of the strand (blue). The 5′-phosphate is depicted as scaled van der Waals spheres and the phosphate atom (purple) is bound in a pocket formed by conserved residues (Asn^13^, Lys^16^, and Lys^26^). DNA at the ds/ss junction is wedged against Arg^53^ and Pro^55^, and the template strand (T) splayed out by Phe^63^ and Phe^64^ with the templating base (yellow) interacting with Phe^64^. (B) NHEJ reactions were performed with PolDom (600 nM) using various substrates formed with the oligonucleotides TTG with NHEJ-D and AAC with NHEJ-D2. When indicated, each of the four NTPs (100 μM) were added in the presence of 1 mM MnCl_2_. (C) Footprinting assays with Polβ (5 μg) or PolDom (5 μg) were conducted as described in Experimental Procedures. BSA (10 μg) was added to the control lane. The substrate was formed with oligonucleotides FP-T, FP-P, and FP-D, depicted on the right. See also Figure S3.

The structural elucidation of a catalytically competent synapsis led us to investigate the molecular features of these polymerases that facilitate DNA binding and extension in a synapsed NHEJ configuration. A common feature of AP and eukaryotic NHEJ polymerases (AEPs and Pol X, respectively) is the requirement for a downstream 5′-phosphate group for DNA binding, indicating that this is an essential requirement for proficient NHEJ. PolDom contacts the recessed 5′ end of the DNA duplex via several residues (Asn^13^, Lys^16^, Lys^26^, Arg^53^, Pro^55^), with residues Asn^13^, Lys^16^, Lys^26^ forming the phosphate-binding pocket (Figure 3A). This positively charged pocket on the surface of PolDom stabilizes the enzyme-DNA binary complex (Figure 3A), and a single point mutation is enough to abolish this interaction (Brissett et al., 2007). This phosphate-binding pocket is specific to AP-NHEJ polymerases and is absent from related replicative primases suggesting that this region evolved specifically for NHEJ (Brissett et al., 2007).

To address if 5′-phosphate (5′-P) binding by each monomer is an absolute requirement for in *trans* extension of short complementary ends, we used a set of 3′-protruding molecules possessing either none or one 5′-P at only one of the two DNA ends (Figure 3B). As expected, extension reactions were specific (in *trans* template directed) and required at least one phosphate group at the 5′ end, with no reaction occurring when 5′-OH was present at both ends (Figure 3B, left panels). When the 5′-P group was in the downstream, template-providing end, there was relevant insertion on the opposite, labeled 3′-protruding end (Figure 3B, central panels). Conversely, when the 5′-P was present in the primer-providing end, insertion was greatly impaired (Figure 3B, right panels). Together, these data indicate that formation of a PolDom synaptic configuration does not strictly require a 5′-P group on both DNA ends. AP-NHEJ polymerases promote strand displacement (Bartlett et al., 2013), ingressing into dsDNA containing no terminal 5′-P to access and bind to an internal phosphate (Brissett et al., 2007). This may be an alternative strategy to stabilize these complexes and also explain the lack of an absolute requirement for a terminal phosphate moiety.

Although a synaptic polymerase arrangement appears to be required to promote break synapsis and extension of DNA overhangs (Brissett et al., 2007), is such a configuration required for extension on a preformed gapped DNA? To address this, we carried out DNase I footprinting with a 1 nt-gapped DNA substrate (Figure 3C). In agreement with previous studies (Pitcher et al., 2007b), DNase I footprinting analysis indicated that a 5′-P group is essential to stabilize the binding of PolDom on a gapped substrate. PolDom’s DNA footprint covers 3 bp on the downstream side, and 5 bp to the primer side therefore, including the templating base, the polymerase binds to 9 nt on the template strand. A similar sized footprint was obtained with human Polβ (Figure 3C), a monomeric polymerase involved in gap filling, suggesting that a single PolDom binds to gapped substrates. Superpositioning of the gapped DNA from the Polβ structure on the PolDom structure further supports this hypothesis (Figure S3A).

### Adjusting the Templating Base for Optimal Binding and Catalysis

**Figure 4:**
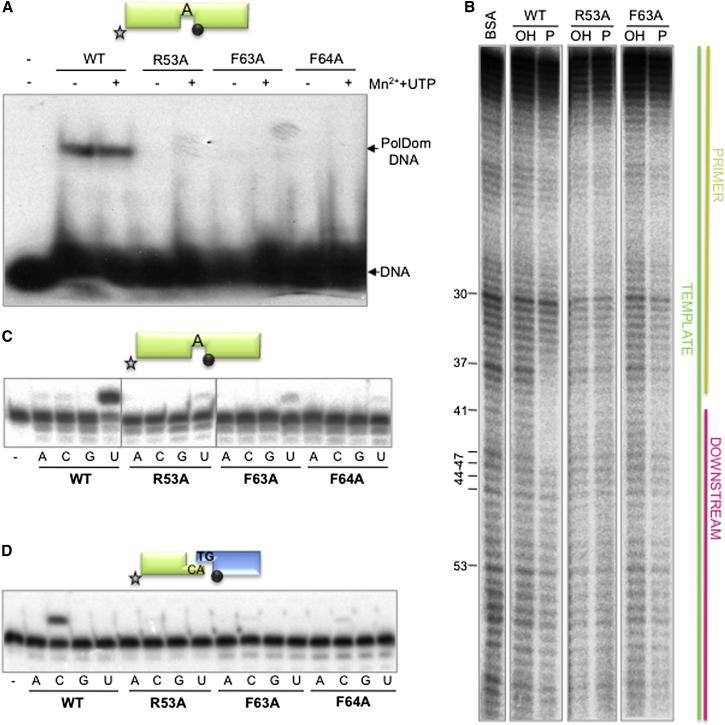
Residues Contacting the Template Strand: Implications for PolDom-Mediated NHEJ Reactions (A) EMSAs were performed for the indicated proteins (200 nM) using a gapped substrate containing the oligonucleotides SP1C, T13C, and DG-P. When indicated, 1 mM MnCl_2_ and/or 100 μM UTP was added. After electrophoresis, the gel was dried and the labeled fragments were detected by autoradiography. (B) Footprinting assays of wild-type or mutant PolDom (5 μg) were conducted as described in the Experimental Procedures. BSA (10 μg) was added to the control lane. The substrate was formed with oligonucleotides FP-T, FP-P, and FP-D, depicted on the right. (C) Gap-filling reactions were performed as described in Experimental Procedures for the indicated proteins (25 nM) using a gapped DNA substrate containing the oligonucleotides SP1C, T13C, and DG-P. When indicated, NTPs were added separately at 10 nM in the presence of 1 mM MnCl_2_. (D) NHEJ reactions were performed with 600 nM of the indicated proteins using a set of DNA substrates formed with the oligonucleotides TG with NHEJ-D and AC with NHEJ-D2. When indicated, each of the four NTPs (100 μM) was added in the presence of 1 mM MnCl_2_. See also Figure S4.

In addition to the phosphate binding residues, other conserved residues (including Arg^53^, Phe^63^, and Phe^64^) also make direct contacts with the single-stranded/double-stranded (ss/ds) DNA junction of each break (Figures 3A and S4), but their specific relevance in end recognition and synapsis remains to be established (Brissett et al., 2007, 2011). Phe^63^ and Phe^64^ are responsible for the major splaying of the templating strand (∼119°) and form (with Arg^53^, Glu^65^, and Pro^55^) a molecular “wedge” that distorts the DNA termini (Figure 3A). Other DNA contacting residues are described in the Supplemental Results (Figures S5 and S6). These intimate contacts appear to play important roles in promoting and maintaining the kinking of the template strand at the ds/ss junction. This orientation is comparable to that observed in Polβ and Polλ complexed with gapped DNA substrates (Figure S3A; Brissett et al., 2007), prompting us to investigate if these residues play more specific roles in the precise alignment of the two ends after productive synapsis. To evaluate their contribution to forming stable complexes on gapped substrates, Arg^53^, Phe^63^, and Phe^64^ were mutated to alanine. The mutants were inefficient at binding to a gapped, 5′-P bearing, DNA substrate as assessed by electrophoretic mobility shift assay (EMSA) and DNA footprinting analysis (Figures 4A and 4B), even in the presence of metal and/or nucleotide. In agreement with this reduction in DNA binding, gap-filling activity of these mutants, including F64A, was barely detectable when compared to wild-type polymerase (Figure 4C). Consistently, NHEJ activity of these mutants was greatly reduced on substrates with a 1 bp complementarity and forming a 1 nt gap (Figure 4D).

**Figure 5:**
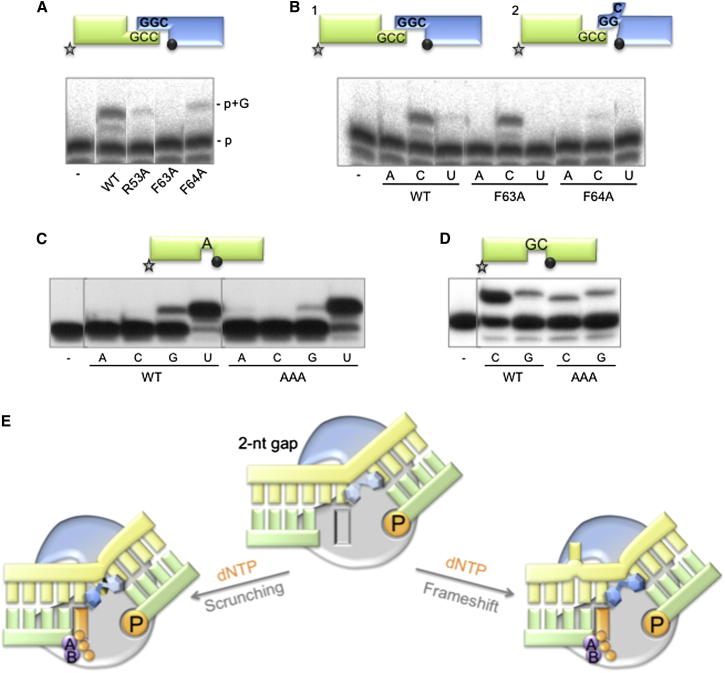
Selecting the Templating Base: Roles of Residues Phe^63^ and Phe^64^ (A and B) NHEJ reactions were performed with 600 nM of the indicated proteins using a set of DNA substrates formed by hybridizing the oligonucleotides CCG with NHEJ-D and GGC with NHEJ-D2. In (A), only GTP (100 μM) was added in the presence of 1 mM MnCl_2_, whereas in (B) the other three nucleotides were added (100 μM). (C and D) Gap-filling reactions were performed as described in Experimental Procedures for the indicated proteins (25 nM) using a gapped DNA substrate containing the oligonucleotides SP1C, T13C, and DG-P (C) or P15, T17, and DG2P (D). When indicated, NTPs were added separately at 10 nM in the presence of 1 mM MnCl_2_. (E) A cartoon showing the dichotomy that PolDom confronts when dealing with gaps longer than 1 nt during NHEJ; the template strand is either “scrunched,” and the gap filled in correctly (left side), or the template strand is dislocated and sequence is lost with the production of frameshifts (right side). The protein is shown as a gray surface with a blue section indicating the approximate position of loop 1, 5′P and incoming nucleotide are colored orange, the two metal ions are shown in purple, and the DNA substrate is shown in yellow (template strand) and green (primer and downstream strands). Phenylalanines Phe^63^ and Phe^64^ are shown as blue hexagons holding the kink in the DNA substrate, indicating with a darker blue color their importance for each reaction. See main text for details.

Next, we tested the mutants on NHEJ substrates having a higher complementarity at the 3′-protrusion (2 dG:dC base pairs) and observed that GTP incorporation was either null (F63A) or barely detectable (R53A and F64A), compared to the wild-type enzyme (Figure 5A). Notably, an unexpected outcome of this experiment confirmed the capacity of the wild-type PolDom to use alternative templating bases during NHEJ, as previously shown in a 2 nt gap context (Pitcher et al., 2007b). In this case, the most favorable connection (2 dG:dC bps) between the two DNA ends would configure a 1 nt gap at each side of the synapsis (Figure 5A, scheme). Thus, the labeled primer (green) should be extended only with GTP. However, when insertion of the other three NTPs was tested, an equally efficient extension with C also occurred (Figure 5B), but no reaction with ATP and UTP was observed, indicating that an alternative templating base (dG) is being used. This templating dG would be available if a single dG:dC base pair is sufficient for the connection, thus configuring a gap of 2 nt (Figure 5B, scheme 1). However, we previously reported that the templating base closest to the 5′P is the preferred one to select an incoming nucleotide, even in the absence of a primer strand (Brissett et al., 2011). Thus, for CTP selection to occur, the preceding templating base (dG) must substitute the favorite dC, but keep the same distance with the 5′P.

A plausible explanation is that dC can be “scrunched” downstream of the polymerization site, awaiting its usage in the next round of nucleotide incorporation (Figure 5B, scheme 2), a model already described for human Polλ in a complex with 2 nt-gapped DNA (Garcia-Diaz et al., 2009). This capacity implies the existence of specific interactions with the scrunched base(s), thus allowing both polymerases to “count”, consecutively reading several templating bases in a gap/NHEJ intermediate. It is likely that residues Phe^63^ and Phe^64^ crucially influence the decision to select the templating base in these situations. Notably, F63A was completely unable to use the favorite templating base (dC) in an NHEJ situation that generates a 1 nt gap (Figure 5A). In contrast, this mutant could insert CTP as efficiently as wild-type (Figure 5B), establishing that the scrunching option is the only operative one and does not require Phe^63^ (scheme 2). As Phe^63^ is crucial for filling a 1 nt gap with no distortions, its irrelevance in a scrunching situation suggests the existence of substitute stabilizing contacts, with the scrunched base as a default. Mutant F64A was only able to catalyze minimal insertion of either GTP or CTP irrespective of the templating base used (dC; Figure 5A), supporting a more general role in orienting any base selected as template.

Loop 1 is also involved in orienting the template strand and essential for synapsis of the two ends (Brissett et al., 2007). We previously reported, using a triple mutant in the apical loop 1 residues (H83A/R84A/S85A or AAA; Brissett et al., 2007), that this loop is crucially important for selecting the templating base in a 2 nt gap. Although the efficiency and fidelity of this mutant for filling in a 1 nt gap was comparable to that of the wild-type PolDom (Figure 5C). However, when presented with a 2 nt gap (Figure 5D), in which PolDom incorporated preferentially CTP, copying the first templating base via scrunching (scheme, Figure 5E), it showed a low level of incorporation of GTP (via dislocation/frameshift, scheme in Figure 5E). The triple mutant maintained the dislocation levels but had a strongly reduced “scrunching” ability (lower CTP incorporation, see Figure 5D). The wild-type PolDom is flexible to choose between these two outcomes on a 2 nt gap, including the formation of a frameshift, by flipping out one of the upstream bases in the template (Figure 5E). In general, choosing the scrunching option will minimize the connection needed and result in the loss of sequences flanking the break.

### Break Protrusions Configured as Primers for Extension during End Synapsis

**Figure 6:**
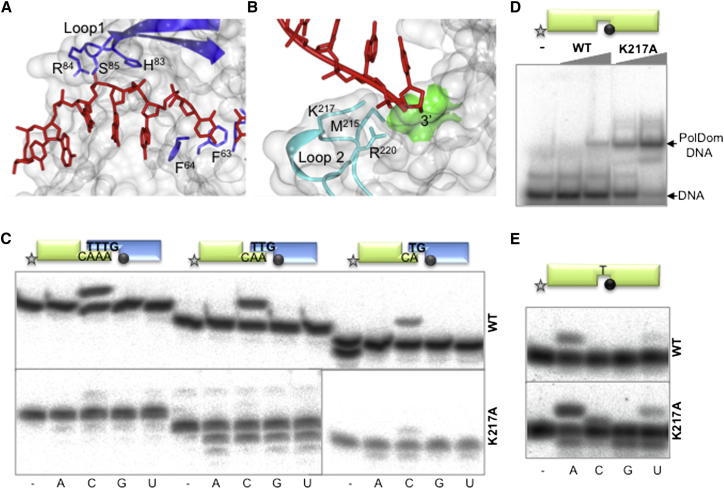
Transition of the Template Strand to Incoming Primer Strand in the Gapped Complex (A) Interaction of loop 1 residues (blue) and their involvement in directing the templating DNA strand (red). A translucent gray surface further depicts the protein solvent accessible surface. His^83^, Arg^84^, and Ser^85^ of loop 1 directs the template DNA that has been splayed by Phe^63^ and Phe^63^ toward the opposing protein monomer. (B) Rotated view of (A) showing the incoming primer strand (red) as it accepted in *trans* into the active site of the opposite protein monomer. Loop 2 and the major interacting residues are colored cyan. A translucent gray solvent accessible surface further depicts the opposing protein monomer. Conserved residues Met^215^, Lys^217^, and Arg^220^ contact the incoming primer as the 3′-OH is stabilized by contacts in the active site (green). (C) NHEJ reactions were performed with 600 nM PolDom using various DNA substrates formed with the oligonucleotides TTTG, TTG, or TG with NHEJ-D and AAAC, AAC, or AC with NHEJ-D2. When indicated, each of the four NTPs (100 μM) were added in the presence of 1 mM MnCl_2_. (D) EMSA assays were performed for the indicated proteins (200 nM) using a gapped DNA substrate containing the oligonucleotides SP1C, T13C, and DG-P. (E) Gap-filling reactions were performed as described in Experimental Procedures for the indicated proteins (25 nM) using a gapped DNA substrate containing the oligonucleotides SP1C, T13C, and DG-P. When indicated, NTPs were added separately at 10 nM in the presence of 1 mM MnCl_2_. See also Figures S5 and S6.

AP-NHEJ polymerases have the capacity to accept and extend an in *trans* “primer” introduced by synapsis with the adjacent break overhang (Brissett et al., 2007, 2011). To understand the molecular basis for this process, we examined the specific contacts made between each polymerase monomer and the incoming strands. This process begins with the kinking of the 3′ template strand from each complex by Phe^63^ and Phe^64^ (Figure 3A). Subsequently, this strand is bound and stabilized by contacts with conserved residues on loop 1 and guided toward the opposing polymerase (Figures 6A, S5, and S6A). Mutation of the apical residues in loop 1 significantly negated end synapsis and extension off these termini (Brissett et al., 2007). The overall conformation of loop 1 does not significantly differ from previous PolDom structures except that His^83^ adopts a different rotamer conformation (Figures 6A and S4). Contacts between loop 1 and the 3′ side of the template strand represent the last major interactions on the overhang’s “journey” away from the binary complex. At this point the 3′ strand makes the transition from a template strand in one complex to becoming a primer strand upon acceptance into the active site of the adjacent binary complex (Figure 2). For this transition to occur, the highly conserved loop 2 (residues 213–224; Figures 2 and 6B), particularly residues Met^215^, Lys^217^, and Arg^220^ make direct contacts with the incoming 3′ strand and channel it toward the neighboring polymerase active site. Additional information about loop 2 contacts is described in the Supplemental Information.

To establish the role played by loop 2 in NHEJ-related DNA recognition processes, we mutated Lys^217^ (K217A) and assayed its activity on NHEJ substrates. K217A mutation significantly affected the ability of the enzyme to promote synapsis (Figure 6C). However, elimination of Lys^217^ increased the ability of the enzyme to bind a gapped-DNA substrate (Figure 6D) and, concomitantly, its activity on these primer-containing substrates was significantly higher (∼50%; Figure 6E). Analysis of the polymerase complex structures suggested that Lys^217^ interacts with the incoming primer strand (Figure 6B). Although no structure of PolDom bound to a gapped DNA substrate is available, it is predicted that steric hindrance between this upstream strand and Lys^217^ would occur thus impairing binding to gapped substrates and impede catalysis. This potentially negative interaction is prevented in K217A, resulting in increased DNA binding and extension activities. These data indicate roles for Lys^217^ and loop 2 in facilitating the more difficult connection of two separated 3′ ends during NHEJ, whereas their intervention on more canonical substrates (e.g., DNA gaps) is not only futile, but may even be detrimental.

### In *trans* Docking of 3′ Hydroxyl of the Incoming Primer in the Polymerase Active Site

**Figure 7:**
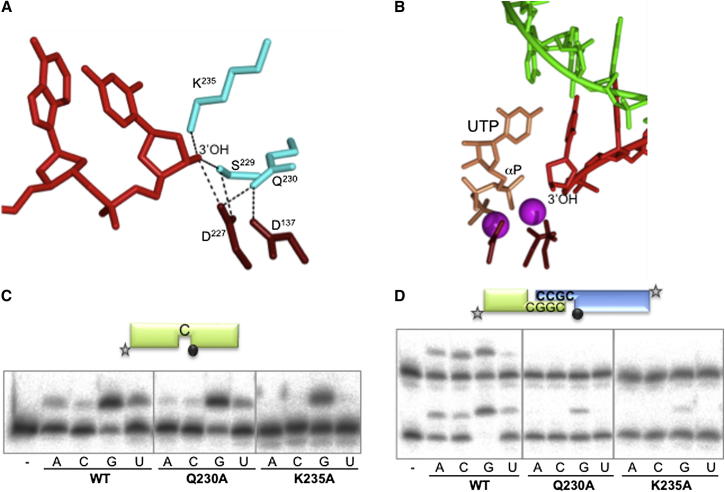
In *trans* Docking of a Primer Terminus in the Polymerase Active Site (A) Interaction and stabilization of the 3′-hydroxyl (3′-OH) of the incoming primer within the active site of PolDom. Residues Lys^235^, Ser^229^, and Gln^230^ (cyan) form a network that interacts with the 3′-OH terminus of the primer strand (red). Two of the catalytic aspartates Asp^137^ and Asp^227^ (brown) are also part of this network. (B) A UTP molecule (tan) and catalytic metal ions (magenta), from a PolDom preternary structure (PDB: 3PKY), were superposed into the active site of the annealed break DNA-PolDom complex. The 3′-OH terminus of the primer strand (red) is within nucleophilic attacking distance of the α-phosphate of UTP, providing compelling evidence that this represents a preternary in *trans* configuration awaiting the arrival of metal ions an incoming base. The templating DNA strand is depicted (green), and the catalytic residues are colored as in (A). (C) Gap-filling reactions were performed as described in Experimental Procedures for the indicated proteins (25 nM) using a gapped DNA substrate containing the oligonucleotides SP1C, T13C, and DG-P. When indicated, NTPs were added separately at 10 nM in the presence of 1 mM MnCl_2_. (D) NHEJ reactions were performed with 600 nM PolDom using a set of DNA substrates formed with the oligonucleotides D3 and NHEJ-D (green, fast running species on the gel) or D4 and NHEJ-D2 (blue, slow running species). Both oligonucleotides were labeled so that primer extension can be observed on both sides of the break at the same time. As indicated, each of the four NTPs (100 μM) was added in the presence of 1 mM MnCl_2_. See also Figure S7.

The structure reveals that the termini of the 3′ overhangs are docked in *trans* into the active sites of the adjacent polymerases. Examination of the catalytic centers has identified several conserved residues that form a network that retains and positions the 3′-OH terminus of the incoming primer strand (Figure 7A). Lys^235^ and Asp^227^ directly contact the primer terminus, and Gln^230^, Ser^229^, and Asp^137^ also form part of this hydroxyl recognition pocket (Figure 7A). To determine if these residues have correctly positioned the 3′OH to allow extension chemistry to occur, we superposed the nucleotide (UTP) and catalytic metal ions from the preternary PolDom-DNA complex (Brissett et al., 2011) into the active site of this “gapped” PolDom-DNA intermediate. As shown in Figure 7B, the hydroxyl moiety is positioned within nucleophilic attacking distance of the α-phosphate of the UTP, suggesting that this structure represents a near catalytically competent ternary-like complex containing an incoming primer strand. However, the 3′ hydroxyl may not yet be in line for nucleophilic attack as previous studies (Brissett et al., 2011) have shown binding of the catalytic metals and an incoming nucleotide facilitates the local rearrangement of loop 2, Asp^139^ and Gln^230^ (Figures S7A and S7F) that likely orientates the primer and α-phosphate for attack. To establish the role of this network of 3′ hydroxyl recognition residues, mutants Q230A and K235A were tested for polymerization on different substrates. Unexpectedly, these mutants maintained a high level of activity on gapped substrates (Figure 7C). The lack of a requirement for primer stabilizing interactions on such substrates is probably due to the established position of the primer strand through interactions with the template strand. To verify this, we tested the mutants in NHEJ assays. Here, the template is a discontinuous strand, and thus the primer needs to be stabilized by the polymerase itself (see scheme in Figure 7D). Both Q230A and K235A showed low levels of nucleotide incorporation compared to the wild-type PolDom, even on complementary substrates (Figure 7D), supporting their proposed role in correctly positioning the 3′ OH moiety for catalysis.

## Discussion

Although AP-NHEJ polymerases are members of the AEP primase family, they function as DNA repair polymerases that are critically required for the recognition and synapsis of double-strand break termini, and subsequent filling in of gaps, to allow restoration of an annealed break in readiness for ligation. These polymerases exhibit a unique variety of activities on different NHEJ substrates including terminal transferase extension on blunt-ended DNA, templated polymerization directed in *cis* on gapped and 5′-protruding substrates (Della et al., 2004; Pitcher et al., 2007b; Bartlett et al., 2013), in *trans* on 3′-protruding substrates (Brissett et al., 2007, 2011), and also a capacity to synthesize across lesions and extend off mismatched primer termini (Pitcher et al., 2007b). Although it has been postulated that AP-NHEJ polymerases, unlike more canonical replicative enzymes, can mediate end extension at the termini of DSBs in *trans*, the details of this reaction mechanism have remained unclear. The molecular analysis presented here provides definitive proof that such an in *trans* extension mechanism does exist, offers insights into how this unconventional polymerase-mediated process occurs, and provides details of how it contributes to the annealing and repair of nonhomologous DSBs requiring end processing. The major mechanistic conclusions derived from this study are summarized in the graphical abstract. Although implicit in this study, this complex is also significant in another respect as it also provides a structural glimpse of a member of the AEP family, that includes eukaryotic replicative DNA primases, bound to a template-primer substrate, thus providing insights into how these essential enzymes bind to and extend a DNA primer strand.

Most DNA polymerases extend in a 5′-3′ direction by nucleotide addition to the 3′ end of a primer strand. However, what happens when a primer strand is unavailable, a scenario that routinely occurs upon formation of particular DSBs. Here, we establish that polymerases themselves can solve this potentially lethal conundrum and still conform to the Kornberg rules for extension, albeit by using an unexpected mechanism. NHEJ polymerases can facilitate the formation of a functional primer-template substrate by docking a primer strand from an adjacent break, to form a gapped intermediate that can now be extended in the canonical way but, significantly, in *trans*. The NHEJ complex presented here highlights the dynamic nature of the end-joining process and illustrates how polymerases can couple end synapsis to catalysis, providing an elegant and simple mechanism to explain how these enzymes are capable of primer extension, even on complex DNA configurations. The structure of an in *trans* DNA configuration has not before been observed, and it establishes another modus operandi for DNA polymerases. There is currently no evidence that higher eukaryotic NHEJ polymerases also work in this way, possibly as they use other proteins (e.g., DNA-PKcs) to facilitate end synapsis. However, it has been reported that yeast NHEJ Pol4 is required for the pairing of 3′ overhangs (Daley et al., 2005b; Daley and Wilson, 2008), suggesting that lower eukaryotes may also require NHEJ polymerases to promote break synapsis under certain circumstances, possibly because they lack additional synapsis factors.

Despite the apparent different origins of the archaeo-prokaryotic and eukaryotic NHEJ polymerases, these two end-joining systems share an unexpected degree of functional and structural commonality. Although architecturally distinct, the catalytic triads of the bacterial and eukaryotic NHEJ polymerases are highly conserved and structurally superposable (Figure S7D), suggesting possible convergent evolution leading to similar catalytic mechanisms. This apparent convergence does not end there because both NHEJ polymerases classes also show a marked preference for the insertion of ribonucleotides over deoxynucleotides. This preference, a possible consequence of the evolution of AP-NHEJ polymerases from the AEP family, reflects a catalytic plasticity that was also acquired during evolution of eukaryotic NHEJ polymerases (Pol X family), such as Polμ (Nick McElhinney and Ramsden, 2003; Ruiz et al., 2003; Martin et al., 2012). Another common characteristic of the AP and eukaryotic NHEJ polymerases is the presence of a binding pocket for the 5′-P group of the downstream DNA strand. This pocket is missing in replicative AEPs from archaea and eukarya but is a major determinant for substrate binding by NHEJ-AEPs and significantly enhances its activity (Pitcher et al., 2007b). Eukaryotic NHEJ polymerases utilize a specific HhH motif to bind this phosphate moiety.

Functional studies on conserved surface loops and flexible elements in Polμ and AEPs have concluded that both classes of NHEJ polymerases rely on mobile structural elements to perform the most critical end-joining activities. For example, PolDom possesses a prominent surface β-hairpin structure (loop 1), which is specific to NHEJ AEPs (Brissett el al., 2007). Conserved residues in loop 1 interact with the 3′ protrusion of NHEJ substrates and orient the synapsis of the ends (Brissett et al., 2007; this study). Mutation of the apical residues of loop 1 to alanine did not affect binding to a primer-containing (gapped) substrate, but abolished the ability of PolDom to form synaptic complexes (Brissett et al., 2007) and, consequently, to catalyze in-*trans*-directed additions. Notably, a functional equivalent loop 1 in Polμ is also required for binding and activity on NHEJ substrates (Juarez et al., 2006), through its function in the stabilization of the synapsis of two DNA ends.

The role of PolDom’s loop 1 in stabilization of the template strand is assisted by two conserved phenylalanines, Phe^63^ and Phe^64^, that maintain the kink in the DNA backbone through stacking interactions with the templating base and the following base, already paired to the 5′-P containing downstream nucleotide. These two amino acids negotiate the selection of the templating base, particularly in cases where more than one candidate exists. It has been shown that, in gapped substrates, PolDom has the ability to dislocate and realign the template, extending the primer by inserting nucleotides complementary to templating bases distal to the primer terminus (Yakovleva and Shuman, 2006; Pitcher et al., 2007b). This behavior stems from the intrinsic capacity of PolDom to dislocate one or more proximal templating bases, generating base substitutions and frameshift deletions. The ability to dislocate and accept distorting nucleotides is important to maximize the opportunities to bridge two protruding 3′ ends with limited complementarity. Human Polμ also has a similar template dislocation activity and an ability to realign mismatched ends (Zhang et al., 2001; Ruiz et al., 2004). When PolDom’s phenylalanines (Phe^63^ and Phe^64^) were mutated to alanine, each mutant displayed different capacities to adjust the templating base in NHEJ reactions: the mutant lacking Phe^63^ was unable to dislocate the first templating base and forced to select the “template scrunching” option. Conversely, the Phe^64^ mutant has poor dislocation activity and was unable to perform correct scrunching of the second templating base. Thus, the presence of these aromatic residues allows PolDom to choose between a number of options, depending on the level of complementarity of the two DNA ends. It endeavors to use less microhomology, when possible, in order to avoid unnecessary loss of sequence, because it is able to correctly polymerize on gaps longer than one nucleotide after bridging. Moreover, the potential to flip out either the first or the second templating base in this context is of great importance in order to accommodate mismatches or damaged bases that cannot be used as templates during NHEJ reactions.

Structural and biochemical studies also implicate loop 1 in this template-dependent decision-making process. In the catalytically incompetent PolDom synaptic structure (Brissett et al., 2007), loop 1 stabilizes an extracyclic base conformation resulting from a frameshift that generated an upstream complementarity. The efficiency of loop 1 mutant to fill in a 1 nt gap was comparable to that of the wild-type PolDom. However, when confronted with a 2 nt gap in which PolDom incorporated preferentially the nucleotide complementary to the first templating base (scrunching) and a low level of incorporation of the second nucleotide (dislocation), the mutant maintained the dislocation levels but had a strongly reduced scrunching ability. This indicates that loop 1 promotes scrunching of the template strand, allowing PolDom to “count” the templating nucleotides one by one. In this regard, PolDom loop 1 is acting like the loop in the thumb subdomain of Pol λ (Garcia-Diaz et al., 2006), an enzyme that, unlike Polμ, also has a “counting ability” when filling in long gaps.

Another mobile loop on PolDom, loop 2, not only plays a direct role in activating the catalytic mechanism via Arg^220^ (Brissett et al., 2011), but also contributes to the stabilization of the two DNA synapsing ends via Lys^217^, which contacts the DNA in the two synaptic complexes obtained to date (Brissett et al., 2007; this complex). Significantly, the contacts established by this residue are similar in the two synaptic complexes, interacting with the primer strand both in the imperfect (Brissett et al., 2007) and fully complementary synapsis (described here), despite the different orientation of the latter. Mutation of this residue suggests that the role of Lys^217^ differs when the repair reaction can be handled by a single polymerase (gapped substrate) or when a synapsis is required (two binary complexes containing 3′ overhangs). In the first scenario, the presence of Lys^217^ is unnecessary or even detrimental for binding to the substrate, whereas in the second scenario it is essential. These findings, along with a comparison of the structural data, indicate that loop 2 configures a binding platform for the acceptance of the incoming primer strand, which is remodeled to the “open” conformation during the assembly of the preternary complex to direct the arrival of the primer terminus into the active site. This flexible structure is designed to function specifically during NHEJ and, accordingly, is only present in NHEJ-related AEPs (Brissett et al., 2007). Additional residues assist the primer strand to reach its final position and, similar to the function of loop 2 in this regard, are dispensable for gap filling but essential for NHEJ of discontinuous ends. Recent studies on the eukaryotic NHEJ polymerases have shown that specific binding to the primer strand is also critical for end-joining to occur but dispensable for gap-filling reactions (Martin et al., 2012), again indicating that significant functional resonances exist between the two classes of NHEJ polymerases.

Although major progress has been made in identifying cellular factors involved in the detection, signaling, and repair of DSBs, relatively little is still understood about the molecular details of how DNA breaks are brought together and processed prior to ligation. Conventional models of NHEJ repair propose that end synapsis is largely dependent on Ku and DNA protein kinase in higher eukaryotes. This may well be the case for bringing the gross ends of DSBs in close proximity, but what factors assist in the alignment of DNA at the extreme termini of DNA breaks? This current study, and others, establishes that NHEJ-processing enzymes, such as DNA polymerases, also play major roles in orchestrating the synapsis of DSBs, particularly of the extreme termini of nonhomologous DNA breaks in a process called microsynapsis. This process is largely driven by the complementarity between the ends of the DSB. If a precise break occurs, the ends are complementary, and therefore it can simply be reannealed back together and religated. However, if the break is imprecise, and has limited homology, then it requires “chaperoning” to assist in the formation of a stably synapsed intermediate, often assisted by microhomology-mediated annealing, that can then be enzymatically processed before ligation. This microsynapsis process is best performed by NHEJ enzymes, exemplified by DNA polymerases in the current study, because these proteins will ultimately dictate how the termini are processed. These proteins have evolved the molecular attributes to recognize the exact structure of DNA ends, and, using this information, they assist in the optimal alignment and annealing of the extreme termini of broken ends, in preparation for enzymatic remodeling, if required, to optimize the breaks for end-joining. The challenge now is to elucidate further structures of NHEJ complexes, containing additional NHEJ proteins and break configurations, to delineate the complete steps that facilitate the coordinated repair of a variety of DSBs by the NHEJ repair machinery.

## Experimental Procedures

### Crystallization of the PolDom-DNA Complex

*Mt-*PolDom was expressed and purified as previously described (Pitcher et al., 2007b). The oligonucleotides used to generate the DNA for crystallization are detailed in the Supplemental Information. *Mt*-PolDom-DNA complex was prepared by incubating the components at concentrations of 300 and 600 μM, respectively, for 45 min at 4°C with added 10 mM MnCl_2_. The protein/DNA mix was then set up as a hanging drop experiment against 200 mM ammonium chloride, 20% w/v PEG 3350 at a ratio of 2:1, and the drops were incubated at 12°C. Crystals were harvested and cryoprotected in reservoir buffer plus 17% ethylene glycol before snap freezing in liquid nitrogen. All data sets were collected at 100K. Single wavelength diffraction data of *Mt*-PolDom-DNA were collected in-house on a Raxis IV++ with a rotating anode X-ray generator RUH3R. The diffraction data were processed with SCALA (Evans, 2006) with additional processing by programs from the CCP4 suite (Collaborative Computational Project, Number 4, 1994). The statistics for data processing are summarized in Table 1.

### Structure Solution and Refinement of a *Mt-*PolDom Annealed Break DNA Complex

The structure of the PolDom-DNA complex was determined by molecular replacement using the program PHASER (McCoy et al., 2007). The crystallographic model of (apo) *Mt-*PolDom (PDB: 2IRU) was used as a molecular replacement search model. Initial refinement was carried out against 95% of the data with REFMAC5 (Murshudov et al., 1997). The remaining 5%, which were randomly excluded from the full data set, was used for cross-validation by calculating the R_free_ to follow the progress of the refinement. The same subset of reflections was used throughout the refinement. Each cycle of refinement was accompanied by manual rebuilding using the program COOT (Emsley et al., 2010). The structure images were prepared with CCP4mg (McNicholas et al., 2011).

### DNA Substrates

PAGE-purified oligonucleotides were 5′ end labeled with [γ-^32^P]ATP by polynucleotide kinase. The oligonucleotides used to generate the DNA substrates are detailed in the Supplemental Information.

### Construction and Purification of *Mt*-PolDom Mutant Proteins

Site-directed mutagenesis (QuickChange, Stratagene) was performed on the overexpression plasmid for *Mt*-PolDom, DNA constructs were sequenced and transformed into *E. coli* B834(DE3)pLysS. Wild-type and mutant *Mt*-PolDom variants were overexpressed and purified as described (Pitcher et al., 2005).

### EMSA and Polymerization Assays

Assays were carried out essentially as described (Pitcher et al., 2007b). EMSAs were employed to analyze the interaction of *Mt*-PolDom with NHEJ intermediates in 50 mM Tris-HCl (pH 7.5), 0.1 mg/ml of BSA, 1 mM DTT, 4% glycerol, 5-nM-labeled DNA, and different concentrations of *Mt*-PolDom or the indicated mutants. After incubation for 10 min at 30°C, samples were resolved by native gel electrophoresis on a 4% polyacrylamide gel (80:1 (w/w) acrylamide/bisacrylamide). For standard (gap-filling) polymerization assays, the incubation mixture contained 50 mM Tris-HCl (pH 7.5), 1 mM MnCl_2_, 1 mM DTT, 4% glycerol, 0.1 mg/ml of BSA, 5 nM gapped DNA, the indicated concentration of NTPs, and either wild-type *Mt-*PolDom or the indicated mutants. After 30 min of incubation at 30°C, reactions were stopped by adding loading buffer and subjected to electrophoresis in 8M urea-containing 20% polyacrylamide sequencing gels. NHEJ polymerization assays were carried out essentially as described above, but using independent DNA template molecules (unlabeled) and short homopolymeric oligonucleotides as a labeled primer. After electrophoresis, unextended and extended DNA primers were detected by autoradiography. Further details are to be found in the Supplemental Information.

### DNA Footprinting Assays

The indicated proteins at the designated concentrations were incubated with 30-nM-labeled gapped substrate in 50 mM Tris-HCl (pH 7.5), 1 mM DTT, 4% glycerol, and 0.1 mg/ml of BSA. After incubation for 10 min at 37°C, samples were treated with 0.03 units of commercial DNase I for 2 min at 37°C. Reactions were stopped with a buffer containing 20 mM EDTA, and the DNA precipitated with 3 M sodium acetate and 100% EtOH, O/N at −80°C. The DNA pellets were washed with 70% EtOH and resuspended in loading buffer (10 mM EDTA, 95% [v/v] formamide, 0.03% [w/v] bromophenol blue, 0.03% [w/v] xylene cyanol), boiled, and subjected to electrophoresis in 8 M urea-containing 8% polyacrylamide sequencing gels. Labeled DNA fragments were detected by autoradiography.

## Figures and Tables

**Table 1:** Crystallographic Data Collection and Refinement Statistics

Data Collection	
Source	In-house rotating anode X-ray generator RUH3R
Space group	P2_1_

**Unit Cell Dimensions (Å)**	

*a*/*b*/*c*	87.58/80.11/118.39
α/β/γ	90.000/111.62/90.000
Wavelength (Å)	1.54
Resolution (Å)	46.06–2.40
Total Number of observations	389,954
Number of unique reflections	58,964
Overall I/(σI)^a^	12.3 (2.0)
Overall completeness (%)^a^	98.5 (96.1)
*R*_*merge*_ (%)^a^^,^^b^	11.7 (82.5)
Multiplicity^a^	6.6 (6.4)

**Refinement**	

Resolution (Å)	37.64–2.40
No. of reflections	55,990
*R*_factor_/*R*_free_^c^^,^^d^	0.1921/0.2418
Contents of asymmetric unit	12 mol (four protein, eight DNA)

**No. atoms**	

Protein	8,680
DNA	1,060
Water molecules	196
Mean B value (Å^2^)	49.81

**Rmsds**	

Bonds (Å)	0.01
Angles (degrees)	1.26

**Ramachandran Statistics**	

Favored regions (%)	94.9
Allowed regions (%)	4.4
Disallowed regions (%)	0.7
PDB accession code	4MKY

a Values for the highest-resolution shell (2.53–2.40 Å) are shown in parentheses.

b *R*_merge_ = Σ_*hkl*_Σ_*i*_|*I*_*i*_– < *I* > |/Σ_*hkl*_Σ_*i*_ < *I* >, where *I*_*i*_ is the intensity of the *i*th measurement of a reflection with indices *hkl* and < *I* > is the weighted mean of the reflection intensity.

c *R*_factor_ = Σ||*F*_o_|–|*F*_c_||/Σ|*F*_o_|, where *F*_o_ and *F*_c_ are the observed and calculated structure factor, respectively.

d *R*_free_ is equal to R-factor for a randomly selected 5% subset of reflections not used in the refinement.

## Accession Numbers

The Protein Data Bank accession number for the structural data reported in this paper is 4MKY.

## Supplemental Information

Document S1. Supplemental Results, Supplemental Experimental Procedures, and Figures S1–S7

Document S2. Article plus Supplemental Information
