# Supplementary material for: Molecular Basis for DNA Double-Strand Break Annealing and Primer Extension by an NHEJ DNA Polymerase
Source: Cell Rep. 2013 Nov 14;5(4):1108–20. doi: 10.1016/j.celrep.2013.10.016 (PMC3898472; doi:10.1016/j.celrep.2013.10.016)
Supplement: Document S1. Supplemental Results, Supplemental Experimental Procedures, and Figures S1–S7 [file mmc1.pdf]

Supplemental Information

Molecular basis for DNA double-strand break annealing and primer extension by a NHEJ DNA polymerase

Nigel C. Brissett, Maria Jose Martin, Edward J. Bartlett, Julie Bianchi, Luis Blanco and Aidan J. Doherty

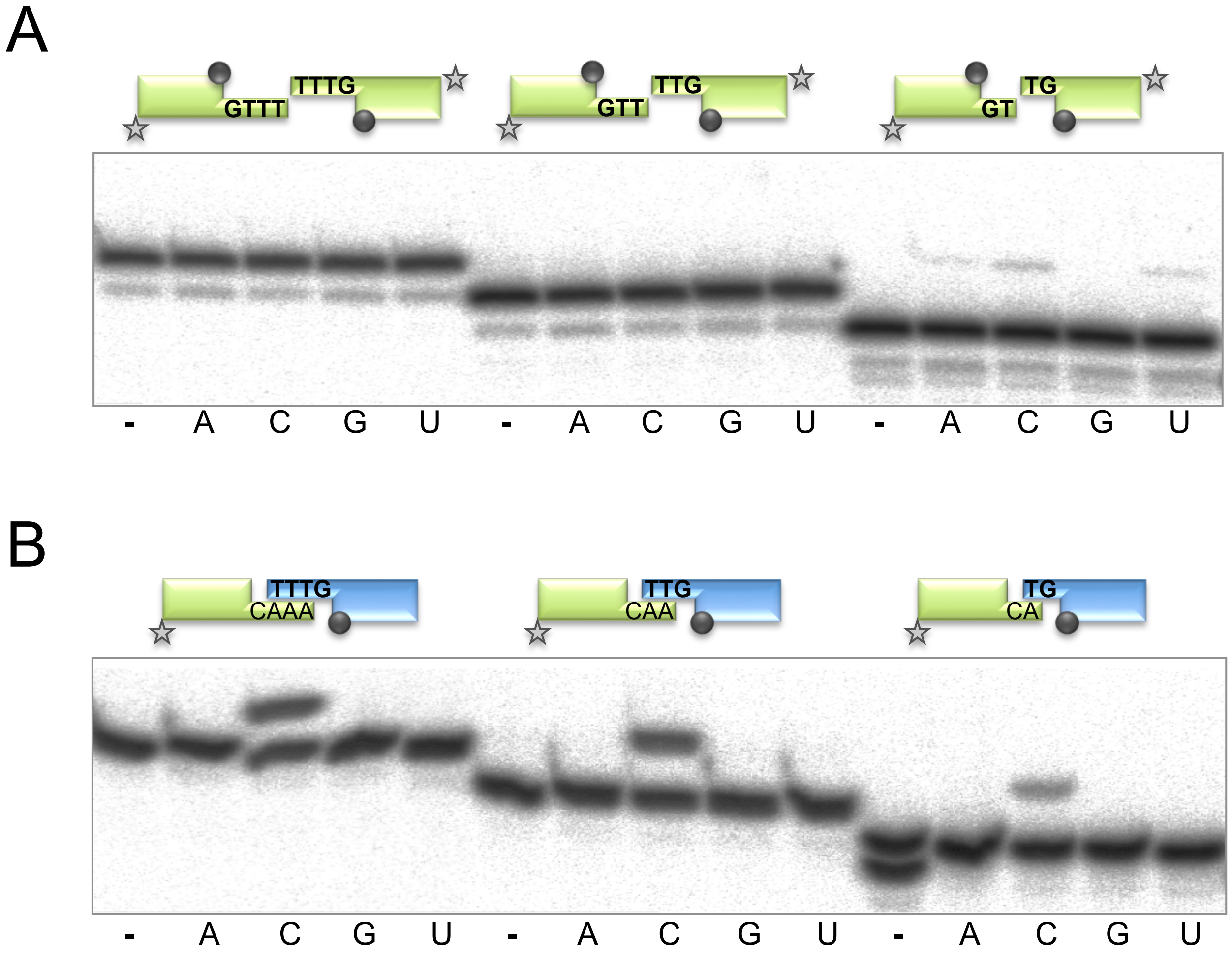
A
GTTT
TTTG
GTT
TTG
GT
TG
- A C G U - A C G U - A C G U
B
TTTG
CAAA
TTG
CAA
TG
CA
- A C G U - A C G U - A C G U

Figure S1

Figure S1 (associated to Figure 1). PolDom-mediated NHEJ synapsis: importance of the length of the 3'-protrusions in the connection.

NHEJ reactions were performed with 600 nM PolDom using various 3'-protruding DNA substrates, formed with the oligonucleotides TTTG, TTG or TG with NHEJ-D. When indicated, each of the four NTPs (100 μM) were added in the presence of 1 mM $MnCl_2$.

(A) PolDom is not able to polymerize on incompatible substrates with long protrusions, probably due to structural constraints. A certain level of error-prone nucleotide incorporation can be observed when the protrusions are only 2-nt long.

(B) When confronted with compatible substrates, PolDom is able to bridge the two ends and trans-polymerize with high accuracy.

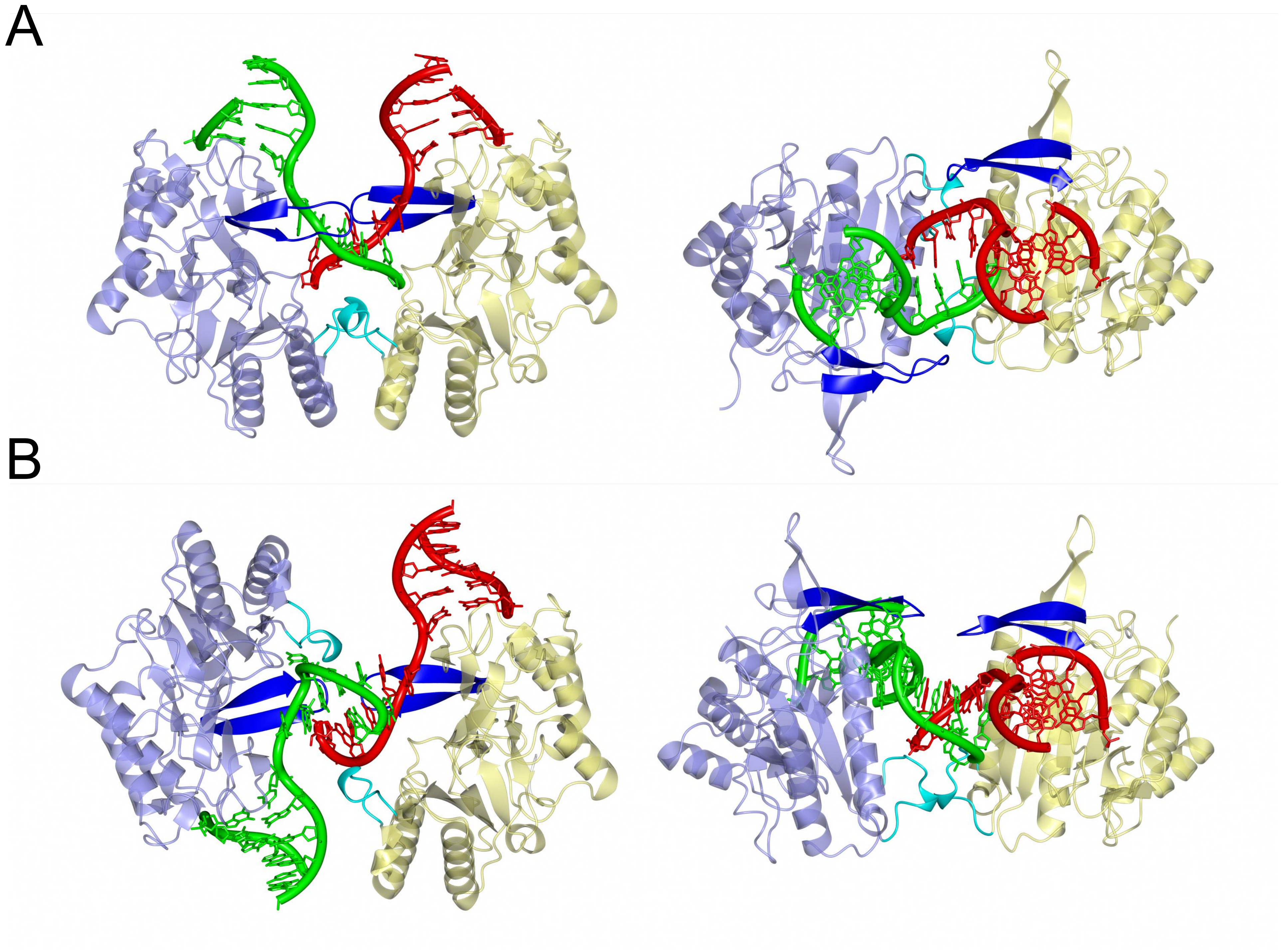

Figure S2

Figure S2 (associated to Figure 2). Comparison of the of catalytically competent and incompetent PolDom-DNA synaptic complexes

Side-on and top-down views of the two PoDom-DNA synaptic intermediates. The protein monomers are coloured blue and yellow, the DNA is coloured red and green to delineate the association with the accompanying protein monomer. Significant structural elements Loop 1 and Loop 2 are coloured blue and cyan respectively.

(A) In the current catalytically competent PolDom-DNA structure (PDBID:4MKY), the protein monomers are in a face to face orientation with the tops of the molecules both facing upwards.

(B) In the previously reported catalytically incompetent synaptic structure (PDBID:2R9L; Brissett et al., 2007), the protein monomers are again in a face to face orientation but the monomers are rotated by 180 degrees relative to one another.

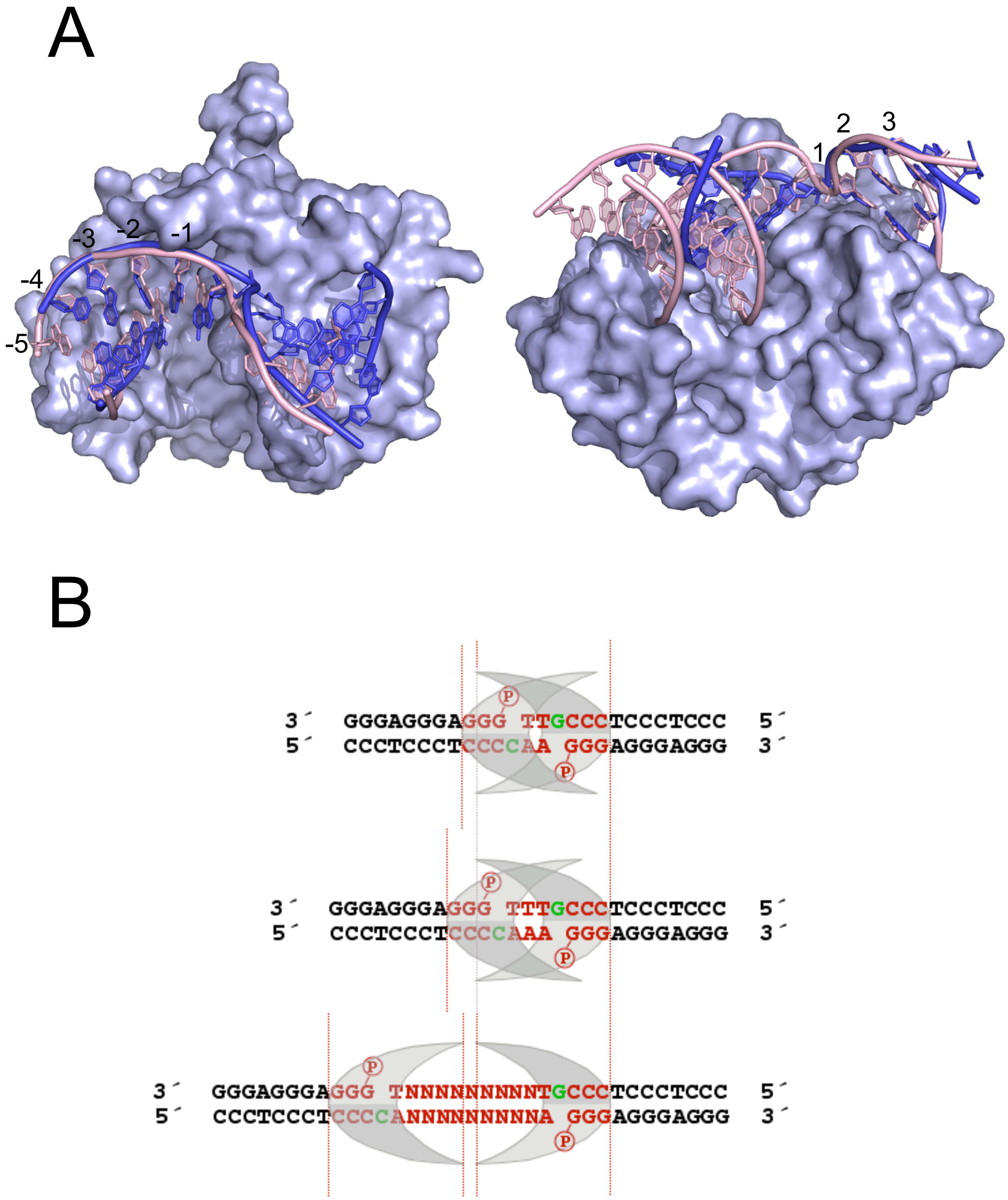
A
-5
-4
-3
-2
-1
1
2
3
B
3´ GGGAGGGAGGG TTGCCCTCCCTCCC 5´
5´ CCCTCCCTCCCCAA GGGAGGGAGGG 3´
3´ GGGAGGGAGGG TTTGCCCTCCCTCCC 5´
5´ CCCTCCCTCCCCAAA GGGAGGGAGGG 3´
3´ GGGAGGGAGGG TNNNNNNNNNTGCCCTCCCTCCC 5´
5´ CCCTCCCTCCCCANNNNNNNNNA GGGAGGGAGGG 3´

Figure S3

Figure S3 (associated to Figure 3). Evidence of the monomeric form of PolDom on an annealed DNA break

(A) Only one PolDom monomer is able to protect 9 nt on the template strand from DNase I cleavage. Superpositioning of a gapped DNA molecule (pink), from a structural complex with Polβ, onto the structure of the PolDom-mediated synapsis (blue) indicates the possible location of the upstream portion of the substrate (-5 base pair, not present in the PolDom structure) that would be covered and footprinted by one PolDom monomer.

(B) The footprint size is compatible with NHEJ reactions involving very short protrusions handled by a dimeric arrangement. A schematic representation of dimeric arrangements of PolDom bridging two DNA ends and the number of nucleotides protected in each case (shown in red). The templating base is shown in green.

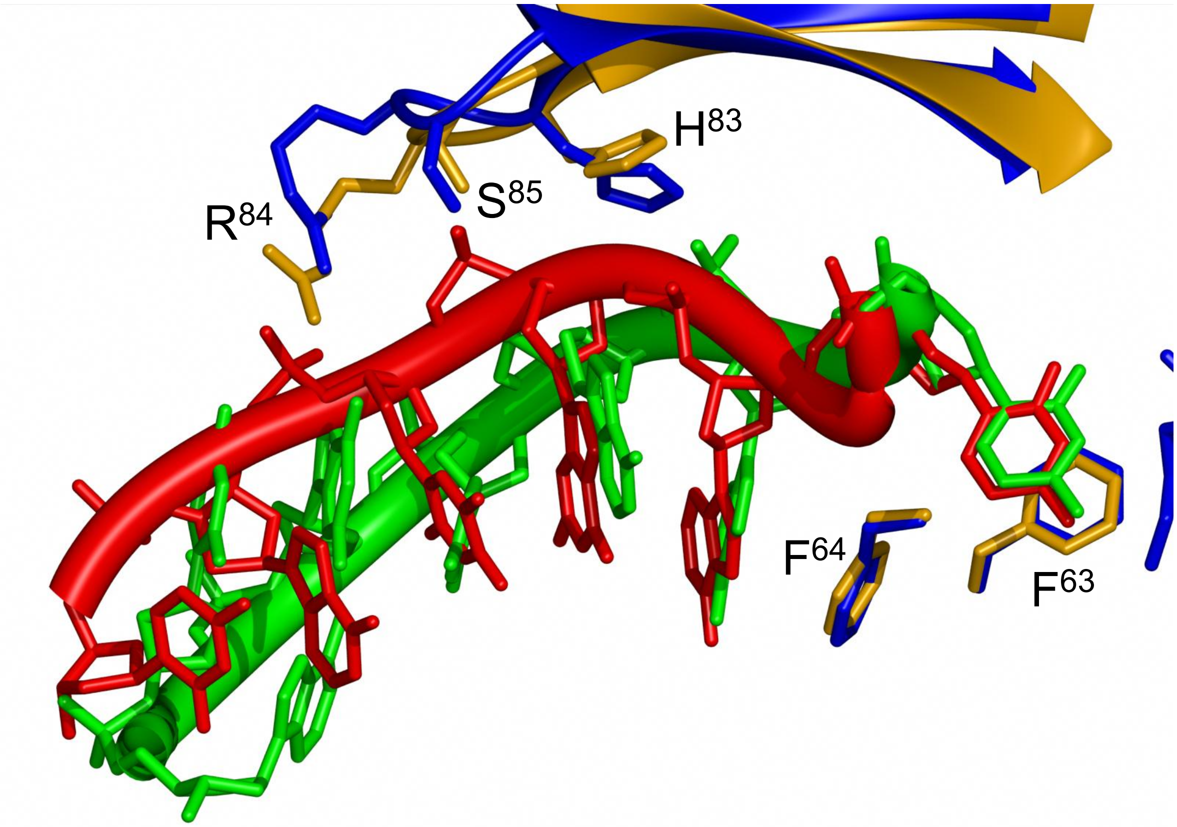

Figure S4

Figure S4 (associated to Figure 4). Residues and structural elements implicated in templating base selectivity and maintaining the kink in the template strand

Influence of Loop 1 residues on the orientation of template strand DNA. Current structure (blue, red; PDBID:4MKY) is superposed with the DNA bound synaptic structure (gold, green; PDBID: 2R9L). Even though there is little perturbation of the residues involved in splaying the DNA at the ds/ss junction (F63/F64), the templating strands from the two complexes follow differing paths. This is due to the contacts with the apical residues of Loop1 (H83, R84, S85).

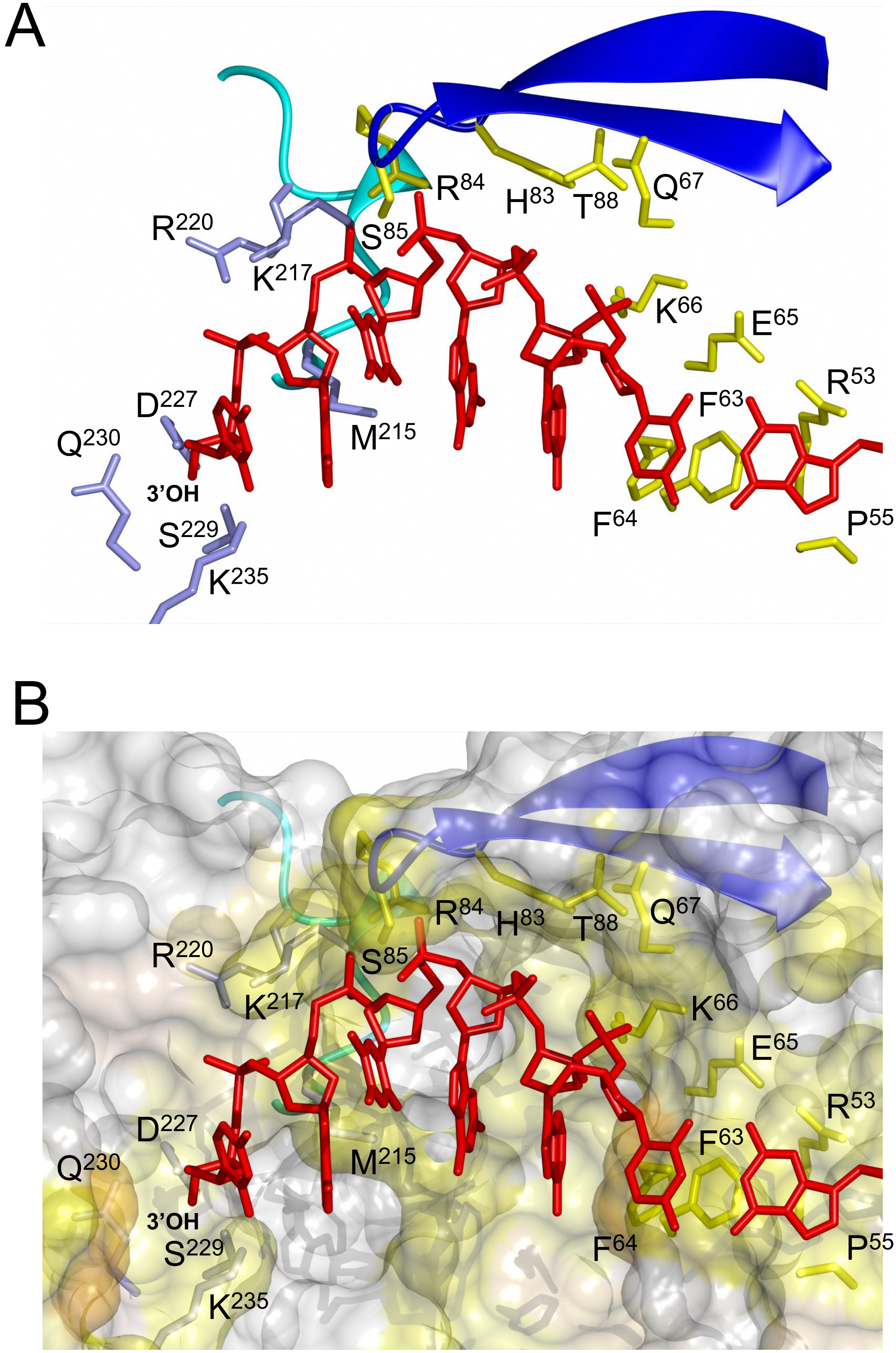
A
R^{84}
H^{83}
T^{88}
Q^{67}
R^{220}
S^{85}
K^{217}
K^{66}
E^{65}
R^{53}
D^{227}
F^{63}
M^{215}
Q^{230}
3'OH
F^{64}
P^{55}
S^{229}
K^{235}
B
R^{84}
H^{83}
T^{88}
Q^{67}
R^{220}
S^{85}
K^{217}
K^{66}
E^{65}
R^{53}
D^{227}
F^{63}
M^{215}
Q^{230}
3'OH
F^{64}
P^{55}
S^{229}
K^{235}

Figure S5

Figure S5. (associated to Figure 6). Loop 1 and Loop 2 protein-DNA contacts direct the transition of DNA from template to primer

(A) Protein contacts of the DNA templating/primer strand. DNA (red) depicted with protein side-chain neighbours that are within 4Å of the strand. Residues are coloured yellow for the protein monomer that binds the ds/ss junction and templating Loop 1 contacts, blue for the protein monomer that accepts the incoming primer strand.

(B) As described above (A) but with a translucent solvent accessible surface. Neighborhood contacts of less than 4Å are tinted yellow.

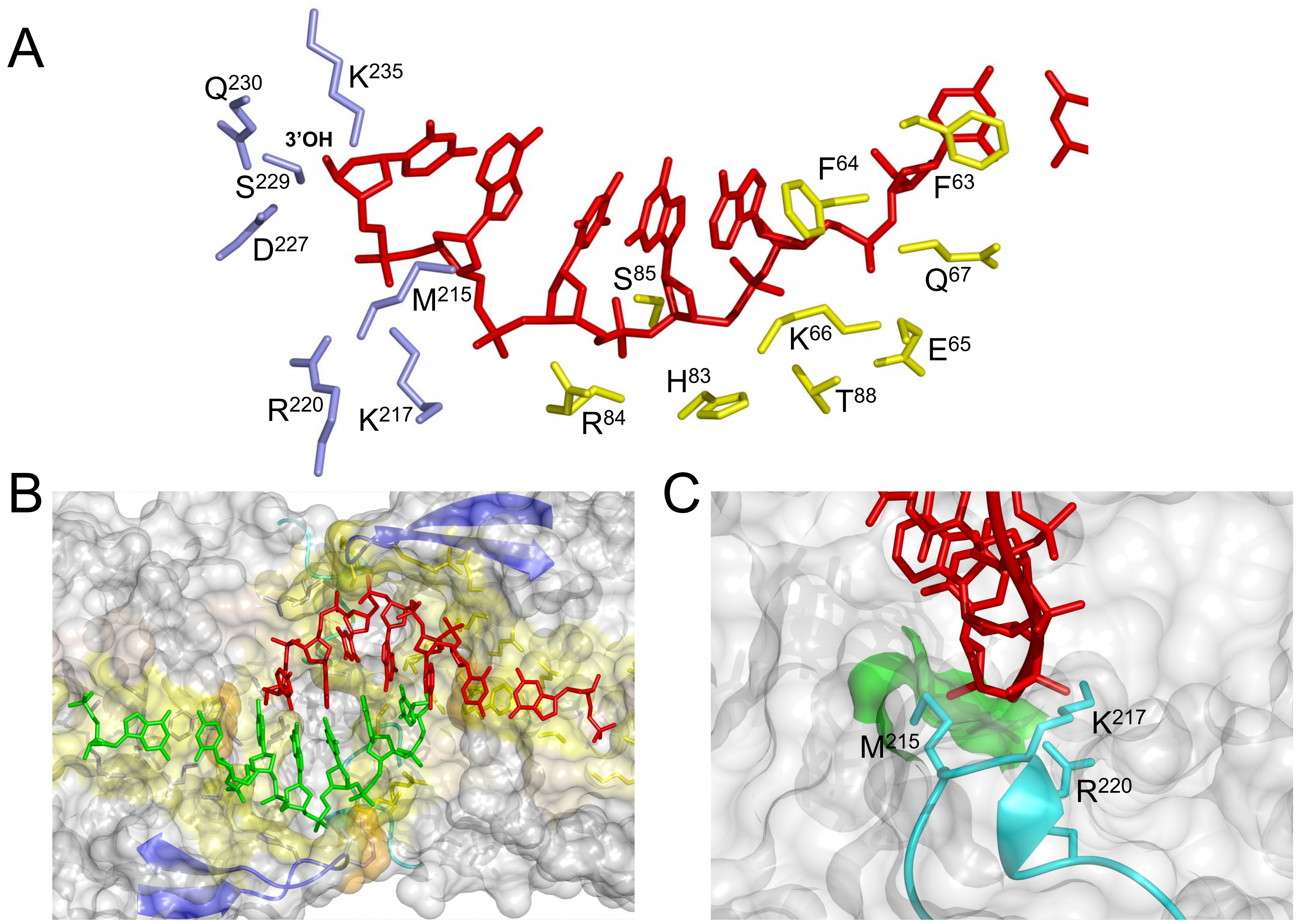
A
K235
Q230
3'OH
S229
D227
M215
R220
K217
R84
H83
S85
K66
T88
E65
Q67
F64
F63
B
C
M215
K217
R220

Figure S6

Figure S6 (associated to Figure 6). Loop 1 and Loop 2 protein-DNA contacts direct the transition of DNA from template to primer

(A) A rotated view of the protein contacts for the DNA templating/primer strand. DNA (red) depicted with protein side-chain neighbors that are within 4Å of the strand. Residues are coloured blue for protein monomer that binds the ds/ss junction and templating Loop1 contacts, yellow for the protein monomer that accepts the incoming primer strand.

(B) A zoomed out view of the template/primer DNA strands contacts with a translucent solvent accessible surface. Neighborhood contacts of less than 4Å are tinted yellow.

(C) A view of the supporting structural role played by Loop 2 (cyan) in guiding the incoming primer strand (red). The translucent solvent accessible surface is coloured green for the area that is in contact with the 3'OH.

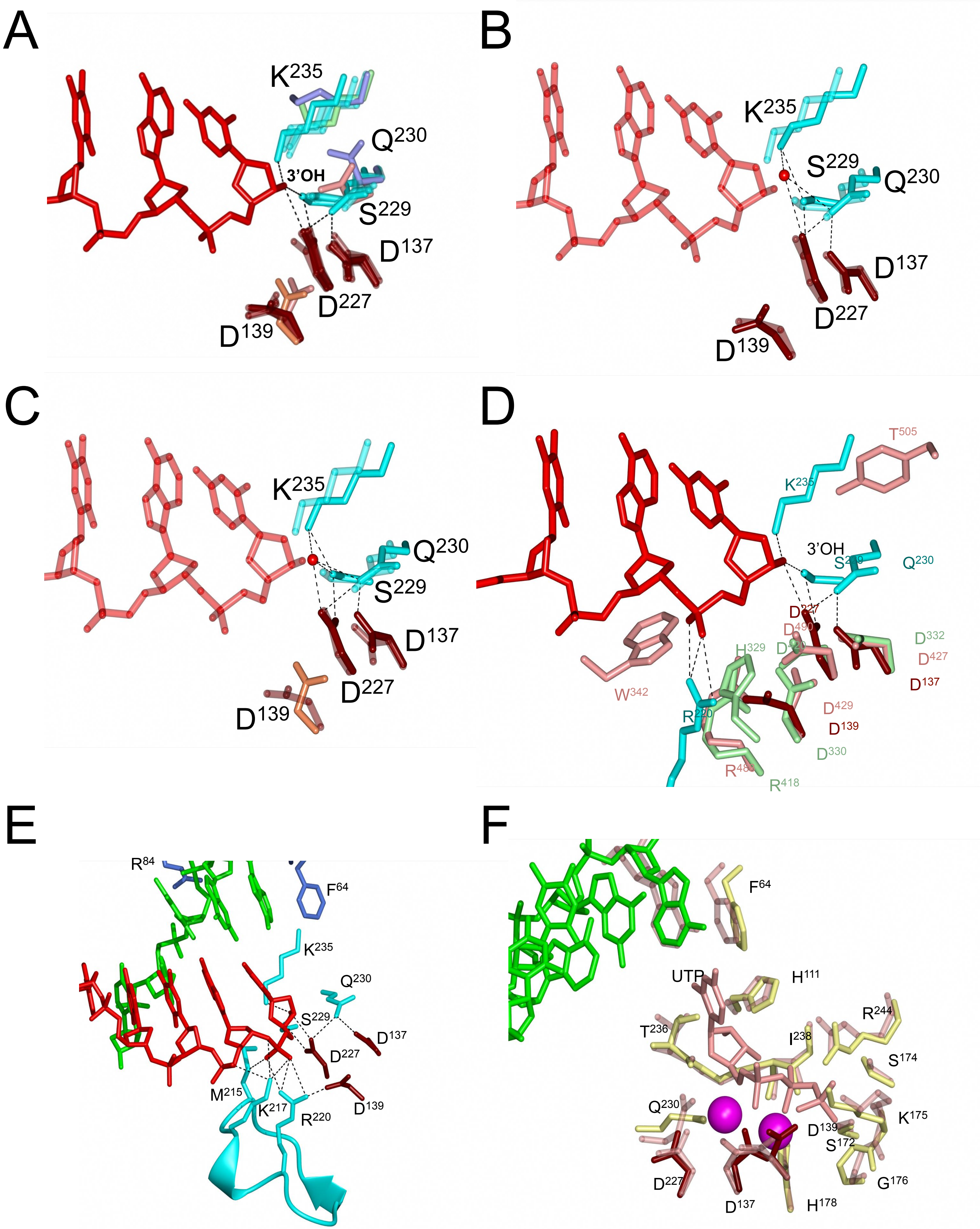
A
K235
Q230
3'OH
S229
D137
D227
D139
B
K235
S229
Q230
D137
D227
D139
C
K235
Q230
S229
D137
D227
D139
D
T505
K235
3'OH
Q230
D332
D427
D137
W342
H329
R220
D429
D139
D330
R418
E
R84
F64
K235
Q230
S229
D137
D227
M215
K217
R220
D139
F
F64
UTP
H111
R244
T236
I238
S174
Q230
K175
D139
S172
D227
D137
H178
G176

Figure S7

Figure S7 (associated to Figure 7). Changes in primer site architecture are driven by status of cofactor binding

(A) All previous PolDom structures (translucent) aligned to the current structure (solid). Major conformational differences are highlighted by colour and solid representation: apo in light green (PDBID: 2IRU), dGTP-bound in light blue (PDBID: 2IRY), GTP-bound in coral (PDBID: 2IRX), and pre-ternary in pink (PDBID: 3PKY).

(B) Position of co-ordinated water (red sphere) in the synaptic structure (solid; PDBID: 2R9L) superposed on the current structure (translucent).

(C) Position of co-ordinated water (red sphere) in the GTP-bound structure (solid; PDBID: 2IRX) superposed on the current structure (translucent).

(D) Superposition onto current structure of catalytic aspartates and primer contacting residues from Polλ (pink; PDBID: 2PFO) and Pol*μ* (light green; PDBID: 2IHM).

(E) Representation of the current structure as a binary (enzyme/substrate) complex.

(F) Conformational changes of ligands involved in nucleotide binding. Overlay of the pre-ternary active site (pink, magenta; PDBID: 3PKY) components onto the current structure (yellow, tan, green).

Supplemental Experimental Procedures

Crystallization studies

Mt-PolDom was expressed and purified as previously described (Pitcher et al., 2007). The oligonucleotides used to generate the DNA for crystallisation were the following: T (5'-GCCGCAGATC-3'), and 5'-phosphorylated D (5'-GCGGC-3'). T/D duplex DNA was prepared mixing equal amounts of the oligonucleotides to give a final solution of 3mM then heating this solution to 95°C and slow annealing over 45 minutes to 4°C in a PCR machine. The crystals grew to an average size of 175mm x 75mm. The crystals belong to space group $P2_1$ unit cell dimensions: a = 87.58Å, b = 80.11Å, c = 118.39Å, $\alpha = \gamma = 90°$, $\beta = 111.62°$. The statistics for data processing are summarized in Table 1.

DNA Substrates

The oligonucleotides used to generate the DNA substrates were the following: homopolymeric ssDNA, PolyA (5'-AAAAAAAAAAAAAAAAAAAAA-3'); for 1-nt gapped substrates, Sp1C (5'-GATCACAGTGAGTAC-3') and 5'-phosphate-containing DG (5'- AGATACACTTCT-3') were hybridized to T13
(5'- AGAAGTGTATCTAGTACTCACTGTGATC-3'); for 2-nt gapped substrates P15 (5'TCTGTGCAGGTTCTT), T17 (5'TGAAGTCCCTCTCGACGAAGAACCTGCACAGA) and
DG2P (5'GTCGAGAGGGACTTCA) were used; for NHEJ substrates, TTTG (5'-CCCTCCCTCCCGTTT-3'), TTG (5'-CCCTCCCTCCCGTT-3'), TG (5'-CCCTCCCTCCCGT-3'), CCG (5'-CCCTCCCTCCCGCC-3') or D3 (5'-CCCTCCCTCCGCGGC-3') were hybridized to NHEJ-D (5'-GGGAGGGAGGG-3') to form template/downstream molecules, and AAAC (5'-GCACTCACGTCCCCAAA-3'), AAC (5'-GCACTCACGTCCCCAA-3'),
AC (5'-GCACTCACGTCCCCA-3'), GGC (5'-GCACTCACGTCCCCGG-3') or D4 (5'-CGCGCACTCACGTCCCCGCC-3') were hybridized to NHEJ-D2 (5'-GGGACGTGAGTGC-3').
For footprinting assays, oligonucleotides FP-T
(5'GGCAGCTTGGATCTTGTCGAAAAACGTCAACATTCGCCTAGGCTTCGGCAATACTGAG GTCTTGTCGAAAAACGTCAACATTGCGTGGCC-3'),
FP-P (5'-GGCCACGCAATGTTGACGTTTTTCGACAAGACCTCAGTAT-3') and
FP-D (5'- GCCGAAGCCTAGGCGAATGTTGACGTTTTTCGACAAGATCCAAGCTGCC-3') were hybridized to form a gapped substrate. NHEJ-D, NHEJ-D2 and FP-D may contain a

phosphate at the 5’-end, as indicated.

EMSA and Polymerization Assays

Assays were carried out essentially as described (Pitcher et al., 2007). EMSAs were incubated in a final volume of 12.5 µl. After incubation, samples were mixed with 3 µl of 30% glycerol and resolved by native gel electrophoresis. For standard (gap-filling) polymerization assays, the incubated (20 µl) reactions were stopped by adding loading buffer (10 mM EDTA, 95% (v/v) formamide, 0.03% (w/v) bromophenol blue, 0.3% (w/v) cyanol blue) and subjected to electrophoresis (Fig. S1).

Supplemental Results

Structure solution and refinement of an *in trans Mt*-PolDom DNA synaptic complex

The structure of the *Mt*-PolDom-DNA complex was determined by molecular replacement using the program PHASER (McCoy et al., 2007). The crystallographic model of (apo) *Mt*-PolDom (PDB id: 2IRU) was used as a molecular replacement search model. A final refined model at 2.4Å resolution, with an R_{factor} of 19.21% and R_{free} of 24.18%, was obtained.

Crystals of *Mt*-PolDom complexed with DNA contained four protein molecules and eight DNA strands in the asymmetric unit, giving a V_M of 2.78 $Å^{-3}Da^{-1}$ corresponding to 55.34% (v/v) water content. The structure comprises amino acid residues 10-293, with no density observed for 9 amino acid residues at the N-terminal end and 7 amino acid residues at the C-terminal end. The terminal two bases from the downstream strand (D) of the duplex are not observed in the electron density. 94.9% of residues in the structure are in the allowed region of the Ramachandran plot with 4.4% of residues in the allowed region and 0.7% of residues are outliers on the plot (Table 1).

The conformational differences between this complex and previously determined PolDom structures were measured by pair-wise alignment methods. The RMSD between apo PolDom and monomer A was 0·55 (over 283 Cα positions) and 0·77 (over 281 Cα positions) for monomer B. The overall structure of the PolDom monomers shows little variation from previously reported structures (PDBID's: 2IRU, 2IRX, 2IRY, 2R9L & 3PKY) with RMSD values of 0.8 or less for all the protein monomers in all combinations. When comparing the current structure to the nucleotide co-crystal structures (PDB: 2IRX, 2IRY; GTP & dGTP bound, respectively) the RMSD's were 0·69 (over 282 Cα positions), 0·59 (over 284 Cα positions) for monomer A, 0·59 (over 282 Cα positions), and 0·76 (over 282 Cα positions) for monomer B. Further comparisons with the PolDom synaptic complex showed RMSD's of 0·64 (over 284 Cα positions), and 0·77 (over 282 Cα positions) for monomers A and B, respectively. Additional comparison with the pre-ternary structure showed RMSD's of 0·71 (over 282 Cα positions), and 0·81 (over 281 Cα positions) for monomers A and B, respectively. Thus, the latest PolDom structure adopts conformations that vary within the complex and closely match apo PolDom for monomer A and the GTP bound form for monomer B. When comparing the

Loop 2 (Figures 2, S2, cyan) conformation of the current structure to those of the previously reported structures, we observed that it is similar to the Apo, NTP-bound co-crystal and synaptic structures, leaving the pre-ternary complex (PDBID: 3PKY) as the only complex with the unique open Loop 2 conformation (Brissett et al., 2011).

Protein-DNA contacts in the annealed break DNA complex

As has been noted previously (Brissett et al., 2007), *Mt*-PolDom interacts with the DNA duplex predominantly via contacts with the recessed 5' phosphate moiety (Asn^{13}, Lys^{16}, Lys^{26}, Arg^{53}, Pro^{55}; the last four being invariant in LigDs; Figs. 2, 3 and S2). There are no significant differences between the three DNA-bound PolDom structures (synaptic, pre-ternary and annealed break), the notable changes mainly arise in the pre-ternary complex (Brissett et al., 2011). Further contacts with the template strand are depicted in figures S4 and S5, most of these are highly conserved among LigD members. The main-chain and side-chain atoms of Lys^{66} are in non-bonded contact to A6 and maintain the templating base in its spatial orientation. Other contacts with the DNA, including Gln^{67} and Thr^{88} are still maintained. As expected, the protein-DNA contacts made by monomer B are almost identical to those for monomer A.

Formation of functional NHEJ complexes on short overhangs: role of 5' phosphate binding and dimeric versus monomeric configurations

Superposition of the gapped-substrate crystallised with Polβ on the structure of the microhomology-mediated synapsis by *Mt*-PolDom shows the possible new location of the upstream portion of the substrate, that would be now covered, and thus footprinted, by one PolDom monomer (Fig. S3A). This footprint size could be compatible with NHEJ reactions involving very short protrusions (Fig. S3B) that could be handled either by a single monomer or a dimeric arrangement as that previously described (Brissett *et al.,* 2007).

3´-protrusions in the template strands become primers during PolDom-mediated end-synapsis

Figure S4 highlights the alternative rotamer conformation that His^{83} adopts, which is different to that observed in all previously *Mt*-PolDom structures. This rotomer brings the Cε1 into non-

bonding contact with OH of Tyr^{90}, also the Nδ1 forms a potential H-bond with Oγ1 of Thr^{88}. In previous PolDom/DNA bound structures, Thr^{88} hydrogen bonds with a backbone phosphate oxygen of the templating strand; in the current structure this hydrogen bond is lost. The backbone carbonyl of His^{83} hydrogen bonds with the phosphate oxygen O1P of templating strand T8. Arg^{84} terminal amide groups interact with the phosphate oxygens O1P of templating strand T8 and A9 (Fig. 6A, S5 & S6A-B). Also, the Oγ of Ser^{85} hydrogen bonds with the phosphate oxygens O1P and O2P of templating strand T8.

The overall topologies of the DNA-bound *Mt*-PolDom complexes are different when considering the complexes that have a potential primer strand. The path that the templating DNA adopts is observed to differ between the annealed break and synaptic complexes. This is despite the residues involved with the splaying of the DNA at the ds/ss junction being in the same orientation for both complexes. The difference in the paths of the two templating strands is due to the interactions with the apical Loop 1 residues (His^{83}, Arg^{84} and Ser^{85}, Fig. S4). As shown in more detail in Figs. S5A and S5B, Loop 1 (coloured blue with yellow side-chains) directs the path of the templating strand and “hands off” the strand to the opposite protein monomer (via Loop 2 coloured cyan with light blue sidechains). As the templating strand is passed to the opposite protein monomer, it now becomes the primer strand as it enters the active site. The opposite protein monomer interacts with the incoming primer via the Loop 2 residues, Met^{215}, Lys^{217} and Arg^{220}. The primer terminus (3’OH) interacts with the active site via interactions with Asp^{227}, Ser^{229} and Lys^{235}. Another view of the side-chains that interact with the template/primer strand is shown in Figure S6A, it is seen that the DNA is in contact with protein most of the time. This is more apparent when viewing the solvent accessible surfaces, the patches of yellow (Figs. S5B and S6B) depicting areas of close contact. Figure S6B depicts how the annealed break is protected from the environment by a combination of Loop 1 and Loop 2 elements that make a continuous protein surface.

Figure S6C depicts a reverse angle view of the incoming primer entering the active of *Mt*-PolDom. The feature to note here is that Met^{215} and Lys^{217} cradle the incoming primer and direct the 3’ terminus into the active site. Previously, we reported that Loop 2 exists as a 3_{10} helix in all of the determined *Mt*-PolDom structures, except the pre-ternary complex (Brissett

et al., 2011) in which the helix unravels and adopts a random coil conformation. This significant conformational change results in Cα position shifts of up to ~6Å, inducing a significant repositioning of two conserved residues, Lys^{217} and Arg^{220}. On comparison, the current Loop 2 conformation is similar to the Apo, NTP-bound co-crystal and synaptic DNA structures. Although it has been shown that Arg^{220} regulates the competency of the active site (Brissett et al., 2011), the importance of the highly conserved Lys^{217} remained uncertain. Notably, this positively charged residue contacts the 3'OH of G13 (template strand) in the PolDom-DNA complex featuring an imperfect synapsis of two DNA ends (Brissett et al., 2007). In the current complex, Loop 2 is also implicated in maintaining the position of the incoming primer in the fully complementary synapsis presented here. This is exemplified by contacts with conserved residues Met^{215}, Lys^{217} and Arg^{220} (Fig. 6B, S6C), where Cγ of Met^{215} makes a non-bonding contact with O1P of C10 from the incoming primer strand. Nζ of Lys^{217} hydrogen bonds with O2P of C10 and O5* of A9 of the incoming primer strand. For Arg^{220}, Nη1 hydrogen bonds with O1P and O2P of C10 whilst Nη2 hydrogen bonds with O2P of C10 of the incoming primer strand (Fig. S6A, S6C).

In trans docking of 3' hydroxyl of the incoming primer in the polymerase active site

The 3' hydroxyl directly interacts with the active site residues Asp^{227}, Ser^{229} and Lys^{235} and indirectly with Gln^{230} (Fig. 7A). Site-directed mutants Q230A and K235A show wild type like activity on gapped substrates but very poor activity on annealed breaks as found in the current structure (Fig 7C & D). The preformed template/primer stabilisation hypothesis explains this effect, but it should also be considered that these residues orientate the primer terminus and keep the 3' hydroxl in a 'stand-by' position prior to catalysis, which is specifically required in NHEJ reactions.

Comparison of this residue network, from previously published structures (Figs. S7A-C), demonstrates that the orientation depends on what moiety occupies the active site at the time. For instance, in the apo structure (Fig. 7A), Lys^{235} points away from the site indicating that this residue has a direct purpose in ligating a hydroxyl moiety in the active site. This is borne out by a water molecule being coordinated by Lys235, and the other residues in the network in the synaptic complex (Fig. S7B) and GTP bound co-crystal (Fig. S7C). This coordinated

water occupies almost the exact position of the 3'-hydroxyl of the incoming primer and is displaced when the primer is bound.

In the dGTP-bound and pre-ternary co-crystal structures (Fig. S7A), we observe Gln^{230} adopting a mm -40 rotamer (as opposed to the mt -30 rotamer observed in the other structures; Emsley et al., 2010). We conclude that Gln^{230} is involved binding/recognition of the incoming NTP as well as orienting the primer terminus.

Figure S7D places the 3'-hydroxyl of the incoming primer in the current structure into context with active site residues from Pol λ and Pol μ. This demonstrates that the incoming primer adopts an orientation that could be tolerated by polymerases from the Pol X family. From this we can conclude that the primer terminus positioning in the current complex is compatible with catalysis.

The current structure represents a binary-type complex (Fig. S7E), as it lacks metal ions and a NTP, and the bound DNA provides both primer and template. In Fig. S7E, the current complex is placed in the same orientation as observed in Fig. 3A of Brissett et al., 2011. Loop 2 and active site residues are the in the same conformation as the synaptic (PolDom-DNA binary) complex. The fact that the Loop 2 conformation doesn't change from this conformation suggests that the primer is oriented prior to binding of the NTP and catalysis. Active site residues, in general, maintain their conformations when NTP is bound (Fig. S7F). Only Lys^{175}, Arg^{244} (triphosphate tail binding), Asp^{139} (catalytic metal binding) and Gln^{230} (NTP binding/recognition) have altered conformations.

Supplemental References

Brissett, N.C., Pitcher, R.S., Juarez, R., Picher, A.J., Green, A.J., Dafforn, T.R., Fox, G.C., Blanco, L., and Doherty, A.J. (2007). Structure of a NHEJ polymerase-mediated DNA synaptic complex. Science 318, 456-459.

Brissett, N.C., Martin, M.J., Pitcher, R.S., Bianchi, J., Juarez, R., Green, A.J., Fox, G.C., Blanco, L., and Doherty, A.J. (2011). Structure of a preternary complex involving a prokaryotic NHEJ DNA polymerase. Mol. Cell 41, 221-231.

Emsley, P., Lohkamp, B., Scott, W.G., and Cowtan, K. (2010). Features and development of Coot. Acta Crystallogr. D Biol. Crystallogr. 66, 486-501.

McCoy, A.J., Grosse-Kunstleve, R.W., Adams, P.D., Winn, M.D., Storoni, L.C., and Read, R.J. (2007). Phaser crystallographic software. J. Appl. Crystallogr. 40, 658-674.

Pitcher, R.S., Brissett, N.C., Picher, A.J., Andrade, P., Juarez, R., Thompson, D., Fox, G.C., Blanco, L., and Doherty, A.J. (2007). Structure and function of a mycobacterial NHEJ DNA repair polymerase. J. Mol. Biol. 366, 391-405.
